# A DnaA‐dependent riboswitch for transcription attenuation of the *his* operon

**DOI:** 10.1002/mlf2.12075

**Published:** 2023-06-30

**Authors:** Yuan Yao, Hongwei Sun, Wurihan, Gegeheng, Gezi, Kirsten Skarstad, Lifei Fan, Morigen

**Affiliations:** ^1^ State Key Laboratory of Reproductive Regulation, Breeding of Grassland Livestock, School of Life Sciences Inner Mongolia University Hohhot China; ^2^ Department of Molecular Cell Biology and Department of Microbiology Oslo University Hospital Oslo Norway

**Keywords:** DnaA‐dependent, *Escherichia coli*, *his* operon, riboswitch, transcription attenuation

## Abstract

Transcription attenuation in response to the availability of a specific amino acid is believed to be controlled by alternative configurations of RNA secondary structures that lead to the arrest of translation or the release of the arrested ribosome from the leader mRNA molecule. In this study, we first report a possible example of the DnaA‐dependent riboswitch for transcription attenuation in *Escherichia coli*. We show that (i) DnaA regulates the transcription of the structural genes but not that of the leader *hisL* gene; (ii) DnaA might bind to rDnaA boxes present in the HisL‐SL RNA, and subsequently attenuate the transcription of the operon; (iii) the HisL‐SL RNA and rDnaA boxes are phylogenetically conserved and evolutionarily important; and (iv) the translating ribosome is required for deattenuation of the *his* operon, whereas tRNA^His^ strengthens attenuation. This mechanism seems to be phylogenetically conserved in Gram‐negative bacteria and evolutionarily important.

## Impact statement

Transcription attenuation is a mechanism for transcription termination found in several operons responsible for amino acid biosynthesis. In this study, we report the first possible example of a DnaA‐dependent riboswitch for transcription attenuation. The DnaA‐dependent riboswitch might regulate the *his* operon attenuation by alternative secondary structures of the HisL‐SL RNA, dependent on binding of DnaA to rDnaA boxes with the assistance of tRNA^His^. It appears that this mechanism is preserved across Gram‐negative bacteria in terms of phylogeny and carries significant evolutionary relevance.

## INTRODUCTION

Transcription attenuation is a mechanism for transcription termination found in several operons responsible for amino acid biosynthesis^1^, ^2^. It was assumed^1^, ^3^, ^4^, ^5^ that (i) transcription attenuation is a consequence of the secondary structure of the leader RNA which, in turn, is governed by the progress of ribosome translocation along the RNA. (ii) Ribosome progression depends on the availability of a specific amino acid. When a cell is deficient in that amino acid, the ribosome translating the nascent leader mRNA^6^ is arrested at the tandem codons waiting for the specific aminoacyl‐tRNA^7^. As a result, a proximal RNA stem‐loop structure forms, which inhibits the formation of the attenuator stem‐loop, followed by a series of uridines, thus allowing transcription through the structural genes. When the level of that amino acid is sufficient, the ribosome translates the tandem codons and releases the RNA immediately after the stop codon, which prevents the formation of the proximal stem‐loop and allows that of the attenuator, leading to the termination of transcription. (iii) The process does not necessarily include a regulatory protein^4^. The attenuation mechanism is known to apply to the regulation of the *trp* ^3^, ^8^, ^9^, *phe* ^10^, *thr* ^11^, *leu* ^12^, and *his* ^6^, ^7^, ^13^, ^14^ operons.

The *his* operon is responsible for histidine biosynthesis in enteric bacteria^15^, ^16^. The *Escherichia coli his* operon is located at 44 min on the chromosome and consists of an upstream leader *hisL* gene and eight structural genes in the order of *hisG*, *hisD*, *hisC*, *hisB*, *hisH*, *hisA*, *hisF*, and *hisI* ^17^. The transcription of these genes is under the control first of the *hisL* promoter (*hisL*p), and then the regulatory region for attenuation upstream of the first structural gene, *hisG* ^18^. The *his* operon is primarily regulated through transcription attenuation^19^, for which the His leader RNA, the HisL leader peptide, and the attenuator stem‐loop RNA structure have been proposed to be required^4^. The His leader RNA is 180 nucleotides long and is transcribed from the *hisL*p, terminating at the attenuator^19^. The HisL peptide contains 16 amino acids encoded from the 5′ terminal of the His leader RNA. The presence of seven contiguous histidine codons in the leader peptide sequence^13^, ^14^ suggests that both histidine availability and tRNA^His^ play a role in the control of *his* operon transcription^13^, ^20^. The attenuator consists of a stem‐loop structure comprising a stem of 14 base pairs and a loop of five nucleotides, which forms at the 3′ terminus of the leader RNA, followed by a run of U residues^14^ that facilitate the separation of the RNA molecule from the template, thus preventing further transcription of the structural genes in the *his* operon^4^.

Transcription of the *his* operon is also influenced by global metabolic regulation mediated by the levels of the alarmone guanosine 5′,3′‐bispyrophosphate (ppGpp) in a manner that is dependent on overall amino acid availability^15^, ^16^, ^21^. Under conditions of amino acid starvation, the ppGpp level increases, which stimulates transcription of the *his* operon and vice versa^22^. In *Salmonella typhimurium*, the target of ppGpp‐mediated regulation of *his* operon transcription seems to be a specific sequence in the *his L*p region covering the −10 hexamer and discriminator sequence^21^, ^22^. In addition, tRNA^His^ is highly homologous to the His leader RNA in both sequence and secondary structure. Many proteins that bind to tRNA^His^ can also bind to similar structures in the His leader RNA and regulate *his* operon transcription by favoring either the attenuator or antiattenuator configuration^23^. Additionally, a wild‐type degree of chromosomal superhelicity is required for normal *his* operon regulation^24^, although the mechanism underlying this requirement remains unclear.

DnaA is the initiator protein for chromosome replication in most bacteria, binding specifically to DNA sequences (DnaA‐boxes) present within the replication origin *oriC*, resulting in the local unwinding of double‐stranded DNA at the AT‐rich region^25^. DnaA also acts as a transcription factor, repressing or stimulating the transcription of several genes by binding to DnaA boxes located near gene promoters^26^. A study in *S. typhimurium* concluded that DnaA might indirectly affect transcription of the *his* operon by regulating the dosage of the *his R* gene, rather than directly by acting as a transcriptional regulator for attenuation of the *his* operon^27^. So far, the binding of DnaA to RNA and function of such interaction have not been investigated. In this study, we detected the pattern of DnaA binding to RNA in the wild‐type *E. coli* cells by DnaA‐FLAG‐RIP (RNA immunoprecipitation [RIP]) sequencing. We found that DnaA‐IP RNA was enriched in histidine metabolism, and DNA repair processes (unpublished data).

Riboswitches serving as molecular switches are regions of mRNA that have specific ligand recognition and binding domains^28^, ^29^, ^30^. Riboswitches regulate the expression of genes, the protein products of which are involved in the biosynthesis, transport, or utilization of target metabolites^31^. Riboswitches are widely present in all organisms^32^, ^33^, ^34^ and are known to sense a wide variety of molecules, such as purines and their derivatives, protein coenzymes and related compounds, amino acids, phosphorylated sugars^35^, and inorganic ligands^36^. Additionally, Rho‐dependent transcription termination has been shown to be controlled by riboswitches^37^. In this study, we report the first possible example of a DnaA‐dependent riboswitch for transcription attenuation. We found that a DnaA‐dependent riboswitch might directly regulate transcription attenuation of the *E. coli his* operon via alternative secondary structures of the HisL‐SL (the *hisL* gene and subsequent Stem Loop region) RNA as a result of DnaA binding to DnaA boxes in the leader mRNA (rDnaA boxes).

## RESULTS

### The stem‐loop attenuator region is required for transcription attenuation of the *his* operon and bestows a fitness advantage

**Figure 1:**
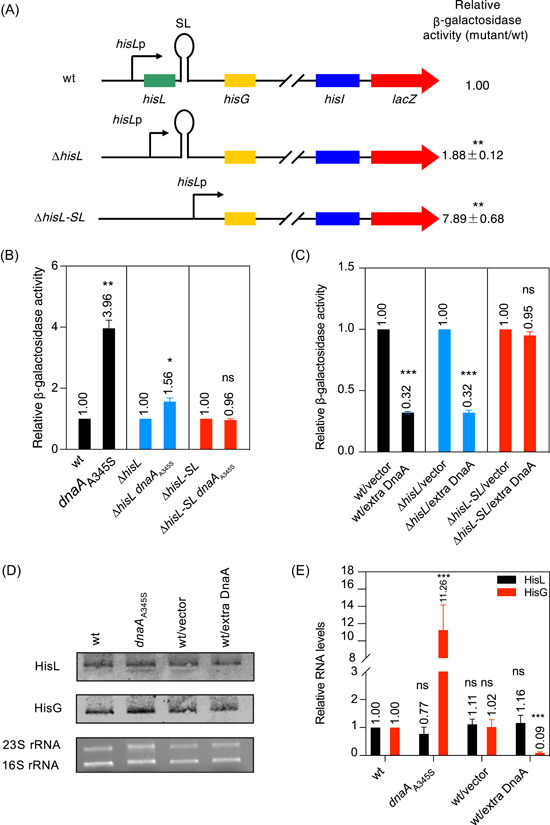
The stem‐loop attenuator region is required for transcription attenuation of the *his* operon and bestows a fitness advantage. (A) Deletion of the *hisL‐SL* region greatly increases *his* operon transcription. Wild‐type (wt) and mutant (∆*hisL* or ∆*his‐SL*) MOR1378 cells were exponentially grown in ABTGcasa medium at 37°C. The *hisL*, *hisG*, *hisI*, and *lacZ* genes are as indicated. Black arrows represent the *hisL* promoter (*hisL*p) and SL represents the stem‐loop region. The relative β‐galactosidase activity in the mutants was compared with that in wt cells as described in the Materials and Methods section. (B) *his* operon transcription is significantly enhanced in the *dnaA*_A345S_ mutant in a *hisL‐SL*‐dependent manner. wt and mutant (*dnaA*_A345S_, ∆*hisL*, ∆*his‐SL*, ∆*hisL dnaA*_A345S_, and ∆*hisL‐SL dnaA*_A345S_) MOR1378 cells were exponentially grown and the relative β‐galactosidase activity in *dnaA*_A345S_ mutant cells was compared with that in control cells as described in the Materials and Methods section. The genotype of each strain is as shown. (C) Excess DnaA represses *his* operon transcription in a *hisL‐SL* sequence‐dependent manner. The relative β‐galactosidase activity was compared between wt and mutant (Δ*hisL* or Δ*his‐SL*) MOR1378 cells, in the presence of *pdnaA*116 (DnaA overproducer) or control vector (pLEX5BA) as described in (A). The genotype of each strain is as shown. (D, E) DnaA regulates the transcription of the structural *hisG* gene but not that of the leader *hisL* gene. (D) wt, *dnaA*_A345S_, wt/control vector, and wt/p*dnaA*116 (DnaA overproducer) MOR1378 cells were exponentially grown in ABTGcasa medium at 37°C. The HisL and HisG RNA levels in total RNA (1 μg/lane) from each strain were analyzed by northern blot using digoxin‐labeled *hisL* and *hisG* probes, respectively. (E) The HisL or HisG RNA level in the same total RNA presented in (D) was determined by RT‐qPCR as described in the Materials and Methods section. The level of each RNA was normalized using the *C*_t_ value corresponding to the *Escherichia coli rplO* gene (internal reference) and calculated relative to that of wt cells. The values are the average of three independent experiments and the standard errors are as presented; ***p < 0.001, **p < 0.01, *p < 0.05, ns, not significant, one‐way analysis of variance.

The His leader RNA and HisL peptide have been suggested to be required for transcription attenuation of the *his* operon^4^. To further confirm this possibility, we constructed strain MOR1378 (Table S1; described in the Materials and Methods section) that carries a *hisI‐lacZ* fusion under the control of the *hisL*p and the *his* regulatory RNA region, *hisL*‐*SL*. Additionally, the *hisL* gene or the complete *hisL*‐*SL* attenuator sequence was removed from the chromosome of MOR1378 cells, resulting in Δ*hisL* or Δ*hisL*‐*SL* MOR1378 cells (Table S1). Transcription attenuation of the *his* operon in these Δ*hisL* and Δ*hisL*‐*SL* cells was calculated as β‐galactosidase activity^38^ under exponential growth at 37°C in ABTGcasa medium containing all the amino acids, except asparagine and glutamine^39^. We found that, compared with that in wild‐type (wt) MOR1378 cells, transcription of the *his* operon was approximately 2‐fold higher in Δ*hisL* cells and nearly 8‐fold higher in Δ*hisL*‐*SL* cells (Figure 1A). These results indicated that (i) the *hisL* gene was required for transcription attenuation of the *his* operon regardless of being a HisL RNA molecule as part of the HisL‐SL attenuator or a peptide and (ii) the presence of the *hisL*‐*SL* region, the product of which had been suggested to form the RNA secondary structure with the stem‐loop attenuator, was an absolute requirement for attenuation, in agreement with previous findings^4^.

Measurements of cell cycle parameters showed that the absence of the *hisL* gene or the *hisL*‐*SL* region resulted in slightly earlier initiation of chromosome replication without disturbing the synchrony of initiation, bigger cells, and slower growth when compared with that of wt cells (Figure S1A,B). These results indicated that the absence of the *hisL* gene or the *hisL*‐*SL* region did not significantly affect the physiology of the cells. Next, to define the possible evolutionary importance of the HisL‐SL RNA‐mediated control of transcription attenuation, the cell fitness measurements were performed (see the Materials and Methods section). For this, four initial mixtures of wt MOR1378 cells and the Δ*hisL* and Δ*hisL‐SL* derivatives were generated in the following proportions: Δ*hisL*/wt cells, 46%:54% or 88%:12%; and Δ*hisL‐SL*/wt cells, 53%:47% or 91%:9% (Figure S1C). After 4 days of growth, no Δ*hisL* cells were present in either Δ*hisL*/wt cell mixture; meanwhile, the proportions of Δ*hisL‐SL* cells were 5% and 29% in the 53%:47% and 91%:9% Δ*hisL‐SL*/wt cell mixtures, respectively (Figure S1C), showing that both mutants had reduced fitness compared with wt cells. After 7 days of growth, no mutant cells were found in any of the four cultures (Figure S1C). These results indicated that the *hisL* and *hisL‐SL* regions bestowed a fitness advantage on the cells and further suggested that the HisL‐SL RNA‐mediated control of the *his* operon may be evolutionarily important.

### Potential DnaA binding sites and predicted secondary structures of the HisL‐SL RNA are conserved in Gram‐negative bacteria

Using the Mfold program (http://www.bioinfo.rpi.edu/applications/mfold/)^40^, we identified three potentially key stem‐loop structures in the *E. coli his* operon that we termed AB, CD, and EF^4^, the last followed by a series of uridines (Figure S2)^4^. The same AB, CD, and EF stem‐loops, followed by uridines were also found in the *his* operons of seven other Gram‐negative species (Figure S2). Sequence alignment showed that the predicted stem domains of these species were highly conserved (Figure S3A). Transcription is likely to terminate at the EF stem‐loop formation as the presence of a row of uridines is predicted to result in the separation of the transcript from the template DNA^4^, ^36^. When the EF attenuator cannot form due to ribosome stalling on the HisL mRNA in response to histidine limitation, transcription continues into the downstream structural genes^1^, ^3^, ^4^, ^5^ and histidine is synthesized, in a process termed “deattenuation.”

Next, we sought to clarify the role of DnaA in transcription attenuation. DnaA can also act as a transcription factor, repressing or stimulating the transcription of genes by binding to DnaA boxes (consensus sequence: 5′‐TTA/TTNCACA‐3′)^25^ located in or near promoter regions^26^. Notably, one study reported that transcription of the *his* operon was increased in several *dnaA* temperature‐sensitive mutants of *S. typhimurium* at the nonpermissive temperature^27^. In this study, we identified four DnaA boxes—two forward and two inverted—in the *hisL‐SL* sequence of *E. coli*. The two forward DnaA boxes, named DnaA box 1 and DnaA box 2, overlapped at positions 46–56 upstream of the transcription start site (+1) of the *hisL* gene; the two reverse DnaA boxes—DnaA box 3 and DnaA box 4—were located downstream of the transcription start site (nucleotides 126–134 and 187–195, respectively). Each DnaA box contained two mismatches relative to the consensus sequence. We named the potential DnaA binding sites in the RNA molecule rDnaA boxes to distinguish them from those in the DNA molecule (Figure S3B). We subsequently identified four putative rDnaA boxes in the HisL‐SL RNA of *Yersinia pestis*, *Trabulsiella odontotermitis*, and *Klebsiella pneumoniae* and three in *Erwinia billingiae*, *Citrobacter freundii*, *Salmonella enterica*, and *Providencia heimbachae* (Figure S2). Interestingly, rDnaA boxes 1, 2, or both are located in front of the AB stem‐loop; rDnaA box 3 is located between the CD and EF stem‐loops; and rDnaA box 4 is found after the EF structure (Figure S2). These findings indicate that potential rDnaA boxes and the predicted secondary structures of the HisL‐SL RNA are highly conserved among the *Enterobacterales* that we investigated. These data further suggest that the DnaA protein may play a crucial and evolutionarily conservative role in transcription attenuation of the *his* operon.

### DnaA regulates transcription of the structural genes but not that of the leader *hisL* gene of the *his* operon

We investigated the possibility that the DnaA protein might play a direct role in transcription attenuation of the *his* operon using the *dnaA*_A345S_ mutant as the DnaA_A345S_ mutant protein has reduced affinity for binding to DnaA boxes^41^. Transcriptional activity of the *his* operon in wt MOR1378 cells and the *dnaA*_A345S_, Δ*hisL*, Δ*hisL dnaA*_A345S_, Δ*hisL*‐*SL*, and Δ*hisL*‐*SL dnaA*_A345S_ derivatives (Table S1) was measured as β‐galactosidase activity in exponentially growing cells, as described above. We found that transcription of the *his* operon in *dnaA*_A345S_ mutant cells was approximately 4–fold higher than that in wt cells (Figure 1B), in line with the findings in *S. typhimurium* ^27^. This result indicated that DnaA functioned as a negative regulator of the transcription of the *his* operon. This effect could result from DnaA either promoting attenuation or repressing the *hisL* promoter through interacting with the rDnaA boxes in the HisL‐SL RNA or the promoter. Transcription in Δ*hisL dnaA*_A345S_ cells was 1.56‐fold higher than that in Δ*hisL* cells (Figure 1B), indicating that DnaA has only a limited effect on *his* transcription in the absence of the *hisL* gene. Interestingly, transcription of the *his* operon in Δ*hisL‐SL dnaA*_A345S_ cells was similar to that in Δ*hisL‐SL* cells (Figure 1B), showing that DnaA function in *his* operon transcription requires the *hisL‐SL* sequence but not the *hisL*p alone. The findings further suggest that the DnaA protein is directly involved in attenuation of *his* operon transcription by interacting either with *hisL‐SL* DNA or its transcript as no peptide product for SL RNA has been identified to date.

To test whether excess DnaA has the opposite effect to mutant DnaA_A345S_ on transcription of the *his* operon (Figure 1B), transcription of the *his* operon was measured in the wt MOR1378 strain and its Δ*hisL* and Δ*hisL*‐*SL* derivatives in the presence of the DnaA expression plasmid p*dnaA*116 or the control vector pLEX5BA^42^ (Table S2). Consistent with the DnaA‐dependent repression seen in wt cells, transcription of the *his* operon was approximately 3–fold lower in the presence of ectopically expressed DnaA relative to that observed with the control vector (Figure 1C). Surprisingly, transcription was 3–fold lower in Δ*hisL* cells in the presence of excess DnaA when compared with the control condition (Figure 1C), suggesting that DnaA‐dependent repression of *his* operon transcription does not require the *hisL* gene or its peptide or RNA; however, DnaA overexpression did not affect *his* operon transcription in the absence of the *hisL*‐*SL* region (Figure 1C). These results indicated that DnaA‐mediated repression was dependent on the presence of the *hisL*‐*SL* region and most likely also the HisL‐SL RNA, as proposed above. The data presented (Figure 1C) are in line with the results described in Figure 1B.

To test whether DnaA exerts a differential effect on transcription of the leader *hisL* gene and the structural genes^17^, we measured the levels of HisL and HisG mRNAs in wt cells, *dnaA*_A345S_ mutant cells, wt cells overexpressing DnaA from the p*dnaA*116 plasmid, and wt cells expressing the control vector pLEX5BA^42^ (Table S2) by northern blot. As shown in Figure 1D, the intensity of the HisL RNA bands was similar in all strains tested, whereas the intensity of the HisG RNA band was stronger in *dnaA*_A345S_ cells and weaker in DnaA‐overexpressing cells relative to that in wt and control plasmid‐expressing cells. As expected, real‐time quantitative‐PCR (RT‐qPCR) analysis identified no significant changes in HisL RNA levels in response to the changes in DnaA availability or activity (Figure 1E, black bars); however, the HisG RNA level was approximately 11‐fold higher in *dnaA*_A345S_ cells and approximately 11‐fold lower in wt cells overexpressing DnaA compared with that in the wt cells (Figure 1E, red bars). These findings indicated that DnaA regulated the transcription of the structural genes in the *his* operon, but not that of the leader *hisL* gene. Furthermore, DnaA is likely involved in the regulation of the *his* operon through interacting with the *SL* regulatory region, which is also supported by the results presented in Figure 1B,C.

### Transcription attenuation of the *his* operon depends on the simultaneous presence of DnaA and the *hisL‐SL* region

**Figure 2:**
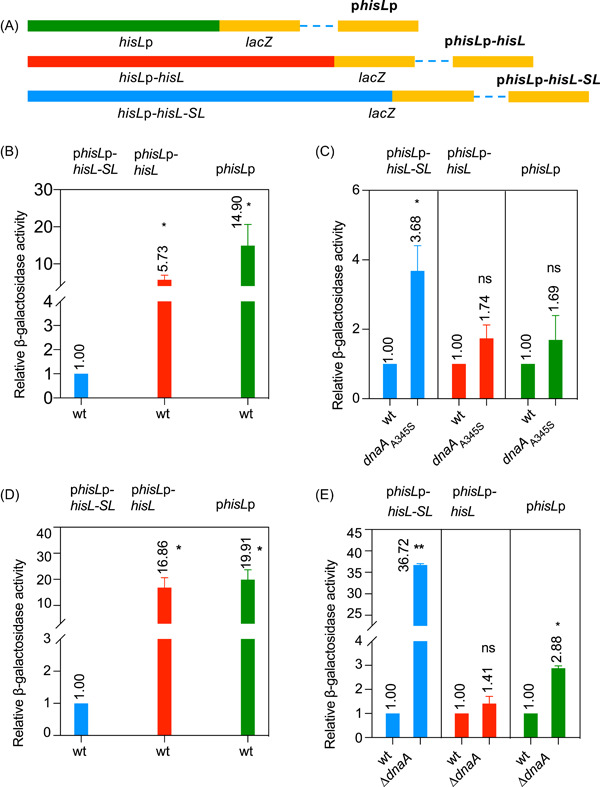
Transcription attenuation of the *his* operon depends on both DnaA and the *hisL‐SL* sequence. The *hisL* promoter (*hisL*p), *hisL*p‐*hisL*, or *hisL*p‐*hisL*‐*SL* fragment was inserted in front of the promoterless *lacZ* gene in the pTAC3953 plasmid as described in the Materials and Methods section, yielding the p*hisL*p, p*hisL*p‐*hisL*, or p*hisL*p‐*hisL*‐*SL* plasmids (A), respectively. In each case, *lacZ* expression was under the control of the *hisL*p, *hisL*p‐*hisL*, or *hisL*p‐*hisL*‐*SL* regulatory element. The relative β‐galactosidase activity in these plasmids was measured in exponentially growing wild‐type (wt) MC4100 cells (B), *dnaA*_A345S_ MC4100 cells (C), wt CM735 cells (D), and *∆dnaA* CM735 cells (E) as mentioned in the legend for Figure 1. The genotype of each strain and the standard deviations are as indicated; **p < 0.01, *p < 0.05, ns, not significant, one‐way analysis of variance.

To clarify the roles of DnaA and the *hisL‐SL* region in *his* operon transcription attenuation, we constructed plasmids where the *lacZ* reporter was placed downstream of *hisL*p, *hisL*p plus the *hisL* gene (*hisL*p‐*hisL*), or *hisL*p plus the *hisL‐SL* attenuator (*hisL*p‐*hisL*‐*SL*) (Figure 2A). Transcription (β‐galactosidase activity) from the plasmids was measured in wt^43^, ^44^ and mutant *dnaA*_A345S_ MC4100 cells, as mentioned above. We found that, in wt cells, transcription was approximately 15‐fold higher with p*hisL*p and 6–fold higher with p*hisL*p*‐hisL* relative to that with p*hisL*p*‐hisL‐SL*, which has the complete *his* operon‐regulatory region (Figure 2B). These results indicated that transcription from the *hisL*p promoter was constitutive in the absence of the *hisL‐SL* attenuator region. Additionally, transcription from p*hisL*p*‐hisL‐SL* in *dnaA*_A345S_ mutant cells was significantly increased relative to that in wt cells; however, the increase was less than 2–fold and not significant in the absence of the *SL* sequence or the *hisL‐SL* region (Figure 2C).

We further examined the transcriptional activity of *hisL*p, *hisL*p‐*hisL*, or *hisL*p‐*hisL*‐*SL* in wt and Δ*dnaA* CM735 cells^45^. In wt cells, transcription from p*hisL*p‐*hisL* and p*hisL*p was approximately 17‐ and 20‐fold higher than that from p*hisL*p‐*hisL*‐*SL*, respectively (Figure 2D), strongly indicating that *his* operon attenuation absolutely requires the *SL* region and, to a lesser extent, also the *hisL* gene. Transcription from p*hisL*p‐*hisL*‐*SL* was approximately 37‐fold higher in Δ*dnaA* mutant cells than in wt cells (Figure 2E). This marked increase in transcription was abolished in the absence of either the *SL* sequence or the *hisL‐SL* region (Figure 2E). Notably, transcription from p*hisL*p in Δ*dnaA* cells was approximately 3–fold higher than that in wt cells (Figure 2E), which may have resulted from the de‐repression of the *hisL*p promoter in the absence of DnaA, given that *hisL*p contains three DnaA boxes (data not shown). These data demonstrated that transcription attenuation of the *his* operon requires both the DnaA protein and the *hisL*‐*SL* sequence.

### The DnaA protein might bind to HisL‐SL RNA and the absence of rDnaA boxes 1, 2, and 3 compromises DnaA binding and subsequently also transcription attenuation

**Figure 3:**
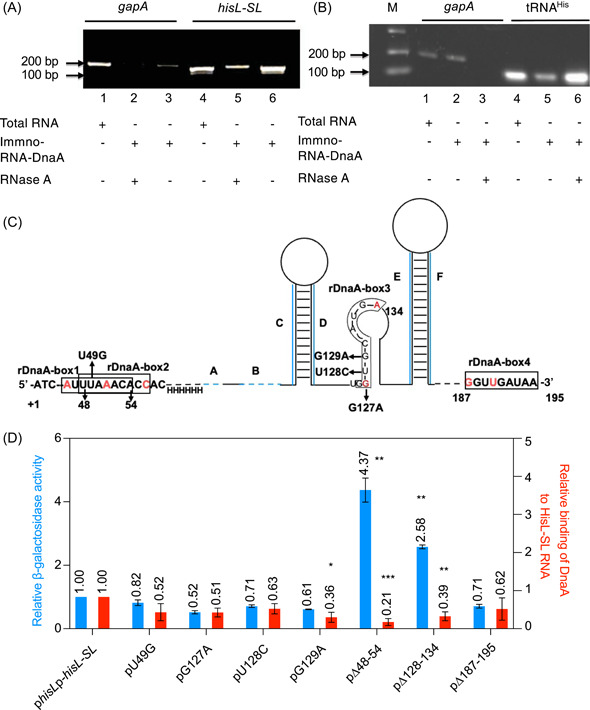
The DnaA protein binds to HisL‐SL RNA and the absence of rDnaA boxes 1, 2, and 3 compromises DnaA binding and subsequently also transcription attenuation. (A, B) The DnaA protein protects the HisL‐SL RNA and tRNA^His^. (A) The *gapA* gene and the *hisL‐SL* fragment were PCR‐amplified using cDNA reverse‐transcribed from DnaA‐RIP RNA as a template and detected on 1% agarose gels. Lanes 1 and 4 were loaded with PCR fragments from total RNA as positive controls; lanes 3 and 6 were loaded with PCR fragments from immunoprecipitated RNA (immno–RNA–DnaA) without RNase A treatment; and lanes 2 and 5 were loaded with PCR fragments from RNA treated with RNase A. The *gapA* gene was used as a negative control as it does not contain a DnaA box. Fragment sizes are as indicated. (B) tRNA^His^ was detected in the immunoprecipitation assay as described in the Materials and Methods section. Lanes 1 and 4 were loaded with PCR fragments from total RNA; lanes 2 and 5 were loaded with PCR fragments from immunoprecipitated RNA (immno–RNA–DnaA) without RNase A treatment; and lanes 3 and 6 were loaded with PCR fragments from RNA treated with RNase A. The DL2000 DNA ladder was used as the size marker (M); the fragment sizes are as indicated in the figure. (C, D) Mutations in the rDnaA boxes of HisL‐SL RNA affect its binding affinity for DnaA and, consequently, transcription attenuation. (C) Deleted nucleotides and point mutations and their positions in the rDnaA boxes in the p*hisL*p‐*hisL‐SL* plasmid are as shown. The sequences of rDnaA boxes 1–4 are boxed; mismatches compared with the consensus sequence are in red. (D) Complementary DNA from exponentially growing MC4100 Δ*hisL‐SL dnaA*‐*flag* cells carrying the p*hisLp*‐*hisL*‐*SL*, pU49G, pG127A, pU128C, pG129A, p∆48‐54, p∆128‐134, or p∆187‐195 plasmid was obtained as described in the Materials and Methods section. The relative binding affinity of DnaA for HisL‐SL RNA was determined as enrichment of the DnaA‐protected RNA molecule by real‐time quantitative‐PCR (RT‐qPCR), as mentioned in the Materials and Methods section. The values were normalized to that of the *rplO* reference gene and the binding affinity of DnaA for the mutated HisL‐SL RNA was calculated relative to that of wild‐type HisL‐SL RNA on the p*hisLp*‐*hisL*‐*SL* plasmid (red bars). Values are averages with the standard error of three individual experiments with two technical replicates. β‐Galactosidase activity from the mutated plasmids in MC4100 cells was measured relative to that in the p*hisLp*‐*hisL*‐*SL* plasmid (blue bars). Values are the averages with the standard deviation of three individual experiments; ***p < 0.001, **p < 0.01, *p < 0.05, one‐way analysis of variance (ANOVA).

The data presented above suggest that DnaA might regulate *his* operon transcription attenuation through binding to HisL‐SL RNA. To further test this possibility, we optimized an RIP assay^46^, and included an RNase A treatment step to remove RNA molecules that were not protected by DnaA. The *hisL‐SL* region was PCR‐amplified using the resultant cDNA as a template and a pair of primers (Figure S8B). DnaA effectively binds to rDnaA boxes‐1,2, so we designed primers to amplify regions containing rDnaA boxes‐1,2 and ensured the shortest fragment (see the Materials and Methods section). The glyceraldehyde 3‐phosphate dehydrogenase A gene (*gapA*) that does not contain a DnaA box served as a negative control. Strong bands for the *gapA* gene (lane 1) and the *hisL‐SL* region (lane 4) were observed for total RNA (Figure 3A). A weak band for *gapA* (lane 3) and a strong band for the *hisL‐SL* region (lane 6) were detected after PCR amplification of immunoprecipitated RNA. Importantly, after treatment with RNase A, a strong DNA band representing the *hisL‐SL* region was detected, whereas no *gapA*‐related product was observed (Figure 3A). These results indicated that the DnaA protein might bind to and protect the HisL‐SL RNA in vivo under physiological conditions. However, no interaction between DnaA and HisL‐SL RNA was found in vitro (Figure S4), suggesting that the binding of DnaA to HisL‐SL RNA may require additional factors. Also, in an in vitro experiment, the DnaA might lose its functions, because we did not examine the activity of DnaA. To further examine if the rDnaA boxes in the HisL‐SL RNA are targets for DnaA binding and whether such binding strengthens the attenuation of *his* operon transcription, we introduced different mutations into the DnaA boxes in the *hisL‐SL* region in the p*hisL*p‐*hisL*‐*SL* plasmid (Figure 2A) by site‐directed mutagenesis^47^. We introduced the U49G mutation into rDnaA box 1/2 (boxes 1 and 2 overlap by seven nucleotides) and the G127A, U128C, and G129A mutations into rDnaA box 3. Additionally, the deletions Δ48‐54, Δ128‐134, and Δ187‐195 were introduced into rDnaA box 1/2, rDnaA box 3, and rDnaA box 4, respectively (Figure 3C). The HisL‐SL RNA transcribed from p*hisL*p‐*hisL*‐*SL* and its mutated derivatives (Figure 3C) in Δ*hisL‐SL dnaA‐flag* cells was immunoprecipitated. The level of binding of DnaA to HisL‐SL RNA was determined as enrichment of the DnaA‐protected RNA molecule using the RT‐qPCR assay. We found that the binding of DnaA to the mutant HisL‐SL_Δ48‐54_ RNA transcribed from the pΔ48‐54 plasmid was approximately 5‐fold lower compared with that for HisL‐SL RNA transcribed from the p*hisL*p‐*hisL‐SL* control plasmid (Figure 3D, red columns). Similarly, the binding of DnaA to mutant HisL‐SL_Δ128‐134_ and HisL‐SL_G129A_ RNA was approximately 3‐fold lower than that to the control RNA. Importantly, these mutation‐related changes were statistically significant (Figure 3D); however, the slight decrease in the level of DnaA binding to mutant HisL‐SL_U49G_, HisL‐SL_G127A_, HisL‐SL_U128C,_ or HisL‐SL_Δ187‐195_ RNA was not significant (Figure 3D). These findings also indirectly demonstrated that the absence of rDnaA boxes 1, 2, and 3 might compromise the binding ability of DnaA to HisL‐SL RNA, further suggesting that rDnaA boxes are targets for DnaA binding.

Next, we asked if the mutations in rDnaA‐boxes disrupted transcription attenuation in the *his* operon. We examined attenuation using the ectopic *his* operon present in p*hisL*p‐*hisL*‐*SL*, its mutant derivatives in strain MC4100, or its *dnaA*_A345S_ mutant, as mentioned above. Transcription from the *his* operon in pΔ48‐54 and pΔ128‐134 was approximately 5‐fold and 3‐fold higher, respectively than that from the p*hisLp*‐*hisL*‐*SL* control (Figure 3D, blue columns), suggesting that rDnaA boxes 1, 2, and 3 are crucial for *his* attenuation. Slight but not significant decreases in transcription were found for the other mutant plasmids (Figure 3D, blue columns). These results showed that the increase in transcription from the *his* operon is strongly associated with the decrease in the affinity of DnaA for the mutant HisL‐SL RNAs. Accordingly, we concluded that direct binding to rDnaA sequences may be one of the possibilities and this binding might function as a switch mechanism for *his* operon transcription attenuation.

Interestingly, in *dnaA*_A345S_ cells, transcription of the *his* operon showed marked mutation‐dependent differences (compare Figure 3D with Figure S5). For the p*hisL*p‐*hisL‐SL* plasmid, *his* operon transcription was approximately 4‐fold higher in *dnaA*_A345S_ cells than in wt cells, while for the Δ48‐54 mutant plasmid, no differences were found between wt and mutant cells (Figure S5). This observation showed that the operon is not sensitive to DnaA in the absence of these boxes, which agrees well with the decreased affinity of DnaA for RNA (Figure 3D) and the increased transcription in wt cells (Figure 3D) when the boxes are deleted. This clearly indicated that rDnaA box 1/2 was a key element in the regulation of *his* operon transcription attenuation. For the Δ128‐134 mutant plasmid in *dnaA*_A345S_ cells, the relative transcription was about 3–fold, which was lower than the 4–fold recorded for p*hisLp*‐*hisL*‐*SL* compared with that in the wt cells (Figure S5), indicating that rDnaA box 3 is also required for *his* operon attenuation. For the pG127A, pU128C, and pG129A plasmids, *his* transcription was approximately 6–fold, 8–fold, and 5–fold higher, respectively, in *dnaA*_A345S_ cells than in wt cells; for the pU49G plasmid, no difference in transcription was found between mutant and wt cells (Figure S5). The differences in transcription imply that the point mutations introduced into the rDnaA boxes might change the affinity of DnaA for the binding sites, given that transcription from these mutant plasmids in wt cells was similar (Figure 3D, blue bars) and that the binding affinity of the mutant DnaA_A345S_ protein for DnaA boxes was low^41^. For the pΔ187–195 plasmid, *his* transcription in *dnaA*_A345S_ cells was 5‐fold higher than that in wt cells (Figure S5), showing that rDnaA box 4 is not important for *his* operon attenuation.

To assess whether mutations in the rDnaA boxes alter the HisL‐SL RNA secondary structure, we used the Mfold program to predict the secondary structures of mutant HisL‐SL RNA (Figure 3C). The results showed that HisL‐SL RNAs with point mutations or the Δ48‐54 and Δ187‐195 deletions retained the AB, CD, and EF stem‐loop attenuators (Figure S6); however, the RNA with the Δ128‐134 deletion retained the EF stem‐loop attenuator, although the sequences of the AB and CD stem‐loops had changed (Figure S6). These results indicated that HisL‐SL RNA harboring these mutations retained its attenuator function.

### Excess tRNA^His^ and the absence of the translating ribosome strengthen transcription attenuation of the *his* operon

**Figure 4:**
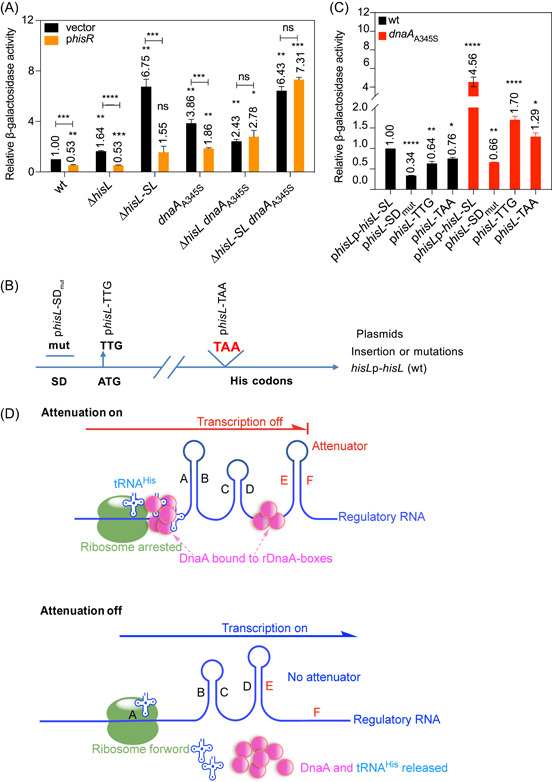
Excess tRNA^His^ and the absence of the translating ribosome strengthen transcription attenuation of the *his* operon. (A) Excess tRNA^His^ strengthens *his* operon transcription attenuation. β‐Galactosidase activity in exponentially growing wild‐type (wt) and derived MOR1378 cells was measured in the presence or absence of excess tRNA^His^ (the p*hisR* plasmid, a tRNA^His^ overproducer) as mentioned in the legend for Figure 1. β‐Galactosidase activity was calculated relative to wt MOR1378 cells expressing the control vector (pACYC177). The values are averages with the standard deviation of three individual experiments; ****p < 0.0001, ***p < 0.001, **p < 0.01, *p < 0.05, ns, not significant, one‐way analysis of variance (ANOVA). (B, C) The absence of the translating ribosome also strengthens translation attenuation of the *his* operon. (B) Mutations in the p*hisL*p‐*hisL*‐*SL* plasmid were introduced by site‐directed mutagenesis, yielding plasmids p*hisL*‐SD_mut_, p*hisL*‐TTG, or p*hisL*‐TAA. (C) Relative β‐galactosidase activity from these plasmids was measured in exponentially growing wt and *dnaA*_A345S_ MC4100 cells as mentioned in the legend for Figure 1. The values are averages with the standard deviation of three individual experiments; ****p <0.0001, ***p < 0.001, **p < 0.01, *p < 0.05, one‐way ANOVA. (D) A model of the DnaA‐dependent riboswitch for transcription attenuation of the *his* operon. The translating ribosome (green) is arrested at DnaA (pink)‐bound rDnaA box 1/2, allowing the formation of the AB, CD, and EF (attenuator) stem‐loops, as indicated, resulting in transcription attenuation. The binding of DnaA to rDnaA box 3 prevents the formation of the alternative DE stem‐loop structure. tRNA^His^ (blue) forms part of the DnaA/rDnaA box complex. When the DnaA protein is released from rDnaA boxes 1, 2, and 3, the translating ribosome covers the A region of HisL‐SL RNA while translating the *hisL* mRNA, allowing the formation of the BC and DE stem‐loop structures; consequently, the transcription machinery reads through the downstream structural genes. Arrows indicate the orientation of transcription.

A decrease in the gene dosage ratio between tRNA^His^ and histidine biosynthetic loci was found to deattenuate transcription of the *his* operon in *S. typhimurum* ^27^. This finding suggests that *hisR*‐encoded tRNA^His^ might negatively regulate *his* operon transcription as greater tRNA^His^ availability would allow the ribosome at the *hisL* gene to progress further without stalling, which, in turn, would lead to transcription attenuation^27^. To test the role of tRNA^His^ in the transcription of the *E. coli his* operon, we cloned the *hisR* gene with its native promoter into pACYC177, yielding the p*hisR* plasmid, a tRNA^His^ overproducer. Transcription of the chromosomal *his* operon in the presence of excess tRNA^His^ was measured as β‐galactosidase activity in wt MOR1378 cells and the Δ*hisL*, Δ*hisL*‐*SL* (Figure 1A and Table S1), *dnaA*_A345S_, Δ*hisL dnaA*_A345S,_ and Δ*hisL*‐*SL dnaA*_A345S_ mutant derivatives (Figure 1B and Table S1). We found that transcription of the *his* operon in wt cells was reduced by 47% in the presence of excess tRNA^His^ compared with the control condition (Figure 4A), in line with previous studies^27^. Similarly, *his* transcription was significantly decreased in Δ*hisL*, Δ*hisL*‐*SL*, and *dnaA*_A345S_ single mutants expressing the p*hisR* plasmid relative to those expressing the control vector (Figure 4A). Interestingly, *his* transcription in the Δ*hisL*‐*SL dnaA*_A345S_ and Δ*hisL dnaA*_A345S_ double mutants was not significantly changed in the presence of the p*hisR* plasmid (excess tRNA^His^) compared with that in the presence of the control vector (Figure 4A). This clearly demonstrates that tRNA^His^‐mediated repression of the *his* operon requires the presence of either the *hisL‐SL* region or the DnaA protein. The results also suggest that DnaA might interact with the HisL‐SL RNA aided by tRNA^His^, and the resultant complex might, in turn, attenuate transcription at the *his* operon. Supporting this conclusion, tRNA^His^ was detected in immunoprecipitation experiments (Figure 3B), indicating that tRNA^His^ might form a complex with HisL‐SL RNA and DnaA.

To test the important role of the translating ribosome on HisL mRNA in the *his* attenuation switch in vivo^4^, we introduced three mutations into the p*hisL*p‐*hisL*‐*SL* plasmid (Figure 2A). As shown in Figure 4B, the p*hisL*‐SD_mut_, p*hisL*‐TTG, and p*hisL*‐TAA plasmids carry a scrambled Shine Dalgarno (SD) sequence, a mutated start codon, and a stop codon inserted in front of the tandem *his* codons in the *hisL* gene, respectively. The scrambled SD site does not allow ribosome loading on the HisL mRNA, while the destroyed initiation codon allows ribosome loading but not the initiation of translation of the HisL mRNA. The inserted stop codon in front of the tandem *his* codons results in the early release of the translating ribosome from the HisL mRNA. All three mutations are expected to result in the formation of the *his* operon attenuator owing to the absence or early release of the translating ribosome. Transcriptional activity (β‐galactosidase activity) of the *his* operon from the plasmids was determined in wt and *dnaA*_A345S_ mutant MC4100 cells. In wt cells, the relative β‐galactosidase activity from the mutated plasmids was decreased compared with that from the wt p*hisL*p‐*hisL*‐*SL* plasmid (Figure 4C), showing that the absence or early release of the translating ribosome strengthens the attenuation of the *his* operon, as previously reported^6^. Notably, the most significant effect on transcription attenuation of the *his* operon was exerted by the scrambled SD site (Figure 4C), indicating that ribosome loading on the HisL mRNA is important for the *his* attenuation switch. As shown in Figure 4C, in *dnaA*_A345S_ cells, the relative β‐galactosidase activity from both the p*hisL*‐TTG and p*hisL*‐TAA plasmids was significantly increased, indicating that the translating ribosome and DnaA act independently on the *his* attenuation switch. Interestingly, the relative β‐galactosidase activity from the p*hisL*‐SD_mut_ plasmid was decreased to 0.34‐fold in wt cells, while it was 0.66‐fold in *dnaA*_A345S_ cells (Figure 4C). These results indicated that DnaA attenuated the transcription of the *his* operon in the absence of loaded ribosome on the HisL mRNA.

## DISCUSSION

### A DnaA‐dependent riboswitch for transcription attenuation of the *his* operon is conserved in bacteria

Riboswitches, found in all organismsorga^32^, ^33^, ^34^, sense a variety of ligands such as purines and their derivatives, protein coenzymes and related compounds, amino acids, and phosphorylated sugars, thereby regulating the expression of associated genes^35^. It has also been shown that riboswitches sense and control Rho‐dependent transcription termination^37^. In this work, secondary structure prediction of the *E. coli* HisL‐SL RNA molecule identified three main stem‐loop structures, termed AB, CD, and EF, the latter followed by a series of uridines (Figure S2), similar to that seen in *Salmonella* ^4^. The same AB, CD, and EF stem‐loops, also followed by uridines, were found to be conserved in Gram‐negative bacteria (Figure S2). Interestingly, four putative rDnaA boxes were identified in the HisL‐SL RNA of *E. coli*, *Y. pestis*, *T. odontotermitis*, and *K. pneumoniae* and three in the RNA of *E. billingiae*, *C. freundii*, *S. enterica*, and *P. heimbachae* (Figure S2). The conserved secondary structures of the HisL‐SL RNA (Figure S2) and positions of the rDnaA boxes suggest that the DnaA protein might play a crucial and evolutionarily conservative role in transcription attenuation of the *his* operon. Indeed, the *hisL* and *hisL‐SL* sequences bestowed a fitness advantage in *E. coli* cells (Figure S1C), indicating that the HisL‐SL RNA‐mediated control for the *his* operon may be evolutionarily important. Furthermore, we found that the DnaA protein might specifically bind to HisL‐SL RNA in vivo (Figure 3A) and mutations in rDnaA boxes 1/2 and 3 significantly decreased the affinity of DnaA for the RNA molecule, leading to deattenuated *his* transcription (Figure 3D). Combined, the data presented in this study suggest that DnaA might facilitate the formation of alternative configurations in HisL‐SL RNA secondary structures by binding to or detaching from the rDnaA boxes, thereby serving as a ligand for the riboswitch, turning on or off attenuation of *his* operon transcription. Unfortunately, we do not have conclusive evidence to indicate that DnaA directly binds to rDnaA boxes. In fact, extracting functional DnaA from in vitro remains challenging and we have explored many different approaches to validate the direct interaction between DnaA and RNA in vitro. However, none of these methods provided stable results, including protein‐centric analysis. Our electrophoretic mobility shift assay (EMSA) results in the supplemental data also failed to show the activity of DnaA. Thus, the following possibilities are still preserved. DnaA might interact with certain RNA‐binding proteins. Some RNAs could form complexes with genomic DNA after transcription and indirectly enrich through the DNA binding activity of DnaA.

### A novel model for the attenuation of *his* transcription by a DnaA‐dependent riboswitch

The current transcription attenuation model assumes that, in the presence of a specific amino acid, transcription will terminate following the formation of the attenuator stem‐loop, whereas in the absence of the same amino acid, the translating ribosome will stall on the leader mRNA^9^, thereby inhibiting attenuator formation and allowing the synthesis of the respective amino acid^1^, ^3^, ^4^, ^5^, ^6^. This process seems to occur in the absence of a specific regulatory protein^4^.

Here, we present a possibility that attenuation of *his* operon transcription depends on both the *hisL‐SL* region and the DnaA protein (Figures 1 and 2). These findings indicated that a close association between DnaA and the *hisL‐SL* sequence was necessary for transcription attenuation of the *his* operon, in agreement with previous results showing that transcription of the *his* operon was increased in several temperature‐sensitive *dnaA* mutants of *S. typhimurium* at the nonpermissive temperature^27^. We further found that, in *E. coli*, DnaA might regulate the transcription of the structural gene *hisG*, but not that of the leader gene *hisL* (Figure 1D,E), by binding to the rDnaA boxes present in the HisL‐SL RNA (Figure 3A,D).

Our data also showed that tRNA^His^ strengthens attenuation of *his* transcription (Figure 4A), in agreement with a previous report indicating that the level of transcription of the *his* operon is inversely correlated with the level of histidyl‐tRNA^20^. Moreover, tRNA^His^‐mediated attenuation was found to require the presence of either the *hisL‐SL* region or the DnaA protein (Figure 4A), while the tRNA^His^ molecule was detected in the immunoprecipitation assay (Figure 3B), suggesting that it forms a complex with HisL‐SL RNA and DnaA (DnaA/HisL‐SL RNA/tRNA^His^ complex), which, in turn, attenuates *his* transcription. In support of this conclusion, the *S. typhimurium* leader mRNA resembles tRNA^His^ in both sequence and secondary structure^23^. Nevertheless, it was found that the DnaA protein might bind to HisL‐SL RNA in vivo (Figure 3A) but not in vitro (Figure S4). Suggesting that, besides tRNA^His^, other factors may also be associated with the DnaA–RNA complex. Indeed, HisG has affinity for tRNA^His^ ^48^, and tRNA^His^‐associated proteins, such as histidyl‐tRNA synthetase, tRNA processing enzymes, ribosomal proteins, and elongation factors, could also interact with the HisL RNA, which has a similar structure to tRNA^His^ ^23^. In line with previous findings^9^, the absence or early release of the translating ribosome was found to strengthen transcription attenuation of the *his* operon, an effect that was independent of DnaA (Figure 4B,C).

In summary, we present a possible novel model for attenuation of *his* operon transcription mediated by DnaA‐dependent alternative configurations in the secondary structure of the regulatory RNA (Figure 4D). When DnaA binds to rDnaA box 1/2 in front of seven His codons, the translating ribosome is blocked^4^ just before the DnaA–RNA complex, leading to transcription attenuation (Figure 3D) resulting from the formation of the AB, CD, and EF (attenuator) stem‐loop structures (Figure 4D). Indeed, the translation of HisL mRNA is required for transcription attenuation in vitro^6^. The binding of DnaA to rDnaA box 3 ensures the formation of the attenuator EF stem‐loop by inhibiting that of the alternative DE stem‐loop (Figure 4D), in agreement with the requirement for the former in attenuation^4^. tRNA^His^ may strengthen the interaction between DnaA and HisL‐SL RNA either through the formation of a HisL‐SL RNA/tRNA^His^ hyperstructure or because DnaA binds to tRNA^His^ as tRNA^His^ and HisL RNA have a similar secondary structure^23^, leading to the formation of a HisL‐SL RNA/DnaA/tRNA^His^ complex (Figure 3B). DnaA may favor binding to double‐stranded rDnaA boxes as rDnaA boxes 2 and 3 in the HisL‐SL RNA form a double‐stranded stem (Figure S7) and tRNA^His^ contains a rDnaA box through which it may base pair with the HisL–SL RNA (Figure S8). Alternatively, as DnaA is released from the HisL‐SL RNA molecule, the translating ribosome can advance, allowing the formation of the alternative BC and DE stem‐loop configurations (Figure 4D). Consequently, the transcription machinery reads through the *his* structural genes. The possible model is mainly supported by the findings that (i) attenuation of the *his* operon requires DnaA, HisL‐SL RNA, and tRNA^His^ (Figures 1, 3, and 4A); (ii) the binding affinity of DnaA for HisL‐SL RNA is inversely associated with transcription of the operon (Figure 3B); and (iii) deattenuation of the operon depends on the translating ribosome (Figure 4B).

## MATERIALS AND METHODS

### Bacterial strains

All the *E. coli* K12 strains (Table S1), plasmids (Table S2), and primers (Table S3) used in this study are listed in Tables S1–S3. The *lacZ* reporter gene was inserted into the MC4100 chromosome behind the *hisI* gene—the last gene of the *his* operon—without disturbing any of the genes in the operon using the method for one‐step inactivation of chromosomal genes, as previously described^49^, ^50^. The MC4100 strain lacks the *lacZ* gene^43^, ^44^. The primer pair H‐*hisI*‐*cat*‐sn and H‐*hisI*‐*cat*‐asn (Table S3) was used to amplify the *cat* gene using pKD3 as a template, yielding a PCR fragment of the *cat* gene flanked at each end by a flippase (FLP) recognition target (FRT) site and a 50‐bp sequence homologous to that of the downstream region of chromosomal *hisI*. The resultant PCR fragment for the *cat* gene was transformed into MC4100 cells with the Red recombinases expressed from the pKD46 plasmid. As a result, the *cat* gene was inserted downstream of the *hisI* gene through Red recombinase‐mediated homologous recombination. The FRT‐flanked *cat* gene was removed from the chromosome using the FLP recombination enzyme expressed from the pCP20 plasmid via FRT site‐specific recombination, leaving an FRT site. Next, the pCE36 plasmid was inserted behind the target site through FLP‐mediated FRT site‐specific recombination between two FRTs: one on the chromosome and one on pCE36. This recombination can insert the *lacZ* reporter gene behind the target gene, *hisI*. pCP20 was subsequently removed by culturing at 42°C, which is the nonpermissive temperature for pCP20 replication, resulting in MC4100 *hisI‐lacZ* (MOR1378). In MC4100 *hisI‐lacZ* cells, the transcription of *lacZ* is under the control of the *hisL* promoter (*hisL*p) and the regulatory elements (*hisL‐SL*), including the *hisL* gene and the downstream region for the stem‐loop attenuator. Additionally, the *hisL* gene or the *hisL‐SL* region in MC4100 *hisI‐lacZ* cells was replaced by the *cat* gene amplified from the pKD3 template with two primer pairs—Fw‐hisL(50)‐F‐cat(20)/Rev‐ds‐hisL(50)‐F‐cat(20) and Fw‐terminator(50)‐F‐cat(20)/Rev‐ds‐terminator(50)‐F‐cat(20) (Table S3) using the method for one‐step inactivation of chromosomal genes^49^. The *cat* gene was then deleted via FRT site‐specific recombination as described above, yielding Δ*hisL* (MOR2062) or Δ*hisL‐SL* (MOR2063) cells. The *dnaA*_A345S_…*cat* allele was P1‐transduced into MC4100 *hisI‐lacZ* and its Δ*hisL* and Δ*hisL‐SL* derivatives as previously described^51^, resulting in MC4100 *hisI‐lacZ dnaA*_A345S_ (MOR1383), Δ*hisL dnaA*_A345S_ (MOR2064), and Δ*hisL‐SL dnaA*_A345S_ (MOR2065) cells. Similarly, the *dnaA‐flag*…*cat* allele was P1‐transduced into MC4100 *hisI‐lacZ* and Δ*hisL‐SL* cells, yielding MC4100 *dnaA‐flag* and Δ*hisL‐SL dnaA‐flag* cells.

### Growth media and conditions

Cells were exponentially grown in ABTGcasa medium^52^. When necessary, 50 μg/ml of kanamycin, 30 μg/ml of chloramphenicol, and 50 μg/ml of ampicillin were added to the medium.

### Plasmid construction

For the construction of the p*hisL*p plasmid, a 641‐bp fragment containing *hisL*p was amplified using the Fw‐hisLp and Rev‐ds‐hisLp primer pair with MG1655 chromosomal DNA serving as a template. The PCR‐amplified *hisL*p fragment was inserted into pTAC3953^53^ at the multiple cloning site (MCS) in front of a promoterless *lacZ* gene using the *Bam*HI/*Hin*dIII restriction sites. To construct p*hisL*p‐*hisL*, a 753‐bp fragment encompassing *hisL*p and the 51 bp of the *hisL* gene was amplified using the Fw‐hisLp and Rev‐hisL‐gene primer pair with MG1655 chromosomal DNA serving as a template. The PCR‐amplified *hisL*p*‐hisL* fragment was inserted into pTAC3953 at the *Bam*HI/*Hin*dIII restriction sites, as described above. To construct the p*hisL*p‐*hisL*‐*SL* plasmid, an 812‐bp fragment containing the *hisL*p, 180 bp of *hisL*, and the *SL* regulatory region was amplified with the Fw‐hisLp and Rev‐hisL‐SL primer pair (Table S3) using MG1655 chromosomal DNA as a template. The PCR‐amplified *hisL*p‐*hisL*‐*SL* fragment was cloned into pTAC3953 at the *Bam*HI/*Hin*dIII restriction sites as mentioned above. In these constructs, *lacZ* transcription was under the control of *hisL*p, *hisL*p*‐hisL*, or the *hisL*p‐*hisL*‐*SL* regulatory region. For the construction of the p*hisR* plasmid, the *hisR* gene with its promoter region (400 bp) was PCR‐amplified using primers Fw‐hisR and Rw‐hisR (Table S3) with MG1655 chromosomal DNA serving as a template, and subsequently cloned into the pACYC177 plasmid at the *Bam*HI/*Hin*dIII restriction sites. For northern blot, the *hisG* gene (897 bp) was PCR‐amplified with primers pTAC3953‐hisG‐F and pTAC3953‐hisG‐R (Table S3) using MG1655 chromosomal DNA as a template, and subsequently inserted into pTAC3953 at the *Bam*HI/*Hin*dIII sites, as described above.

### Site‐directed mutagenesis

As shown in Figures 3C and 4B, mutations were introduced into p*hisL*p‐*hisL*‐*SL* using the QuickChange Site‐directed Mutagenesis Kit and the primer pairs Fw/Rev‐pT49G, Fw/Rev‐pG127A, Fw/Rev‐pT128C, Fw/Rev‐pG129A, Fw/Rev‐pΔ48‐54, Fw/Rev‐pΔ128‐134, Fw/Rev‐pΔ187‐195, Fw/Rev‐pA33T, Fw/Rev‐pTAA, HisL‐SDmut‐F/R, and HisL‐SD‐4Umut‐F/R (Table S3).

### Prediction of the secondary structures of HisL‐SL RNA in bacteria

The DNA sequence (180 bp) of the *E. coli hisL‐SL* region was obtained from the Ecogene library (http://www.ecogene.org). The sequence of the HisL‐SL RNA was then developed from the DNA sequence. Similarly, the sequences of the HisL‐SL RNAs of different bacteria were developed from the *hisL‐SL* DNA sequences obtained from the National Center for Biotechnology Information (NCBI) nucleotide database (https://www.ncbi.nlm.nih.gov/nucleotide/). The secondary structures of the HisL‐SL RNA molecules were predicted using the Mfold program (http://unafold.rna.albany.edu/?q=mfold/RNA-Folding-Form)^40^. Default settings were applied and the folding temperature was set at 37°C.

### β‐Galactosidase assay

β‐Galactosidase activity was determined as previously described^38^. Cells were exponentially grown in ABTGcasa medium at 37°C and sampled at OD_450_ = 0.1, 0.2, 0.3, 0.4, and 0.5. A 1‐ml aliquot of cells at each OD value was mixed with 100 μl of toluene, vortexed vigorously to permeabilize the cells, and then stored at 4°C overnight. The next day, 200 μl of the aqueous phase of each sample was mixed with 1 ml of 0.66 mg/ml 2‐nitrophenyl β‐d‐galactopyranoside (ONPG) dissolved in Z‐buffer and the mixture was incubated at 30°C. The reaction was stopped by adding 500 μl of 1 M Na_2_CO_3_, following which the absorbance of the products at OD_420_ was measured using a microplate reader (Bio Tek). β‐Galactosidase activity was calculated using the formula 1000 × OD_420_/0.2 × *T* × OD_450_, where *T* represents the reaction time (min).

### RIP

#### Cross‐linking

Cells of the MC4100 *dnaA*‐*flag* strain and those of its derivatives were exponentially grown in ABTGcasa medium with the appropriate antibiotics at 37°C (OD_450_ = 0.4–0.5). A total of 100 ml of cells was harvested by centrifugation and then placed at room temperature (RT) for 10 min. The cells were resuspended in 1% formaldehyde and 10 mM sodium phosphate, pH 7.6, and incubated at RT for 15 min, with occasional mixing. Then, 2.5 M glycine (final concentration: 125 mM) was added to the cell mixture, followed by incubation at RT for 5 min, with mixing every 2 min, and subsequently placed on ice for 5 min. The cells were collected by centrifugation at 6000 rpm for 8 min at 4°C, washed with 25 ml of ice‐cold phosphate‐buffered saline (PBS), resuspended in 1 ml of PBS, transferred into a 1.5‐ml tube, collected again, resuspended in 0.5 ml of lysis solution (10 mM Tris‐HCl pH 8.0, 50 mM NaCl, 10 mM ethylenediaminetetraacetic acid [EDTA] pH 8.0, 20% sucrose), and incubated at 37°C in a water bath for 15 min with mixing every 3 min after the addition of lysozyme (final concentration: 10 mg/ml) and phenylmethylsulfonyl fluoride (PMSF) (final concentration: 1 mM). Next, 0.5 ml of 2× radioimmunoprecipitation assay (RIPA) buffer (100 mM Tris‐HCl pH 8.0, 300 mM NaCl, 2% NP‐40, 1% sodium deoxycholate, 0.2% SDS) and PMSF (final concentration 1 mM) were added, followed by incubation on ice for 15 min with mixing every 3 min. The cells were further lysed by repeated freezing and thawing cycles and the supernatant was collected. Then, 50 and 900 μl of the supernatant were separately transferred into two new microcentrifuge tubes as samples for “total RNA (INPUT) extraction” and “immunoprecipitation (IP),” respectively. To remove any residue remaining in the supernatant, the sample was centrifugated twice at 14,000 rpm for 15 min at 4°C. In parallel, EZView Red Anti‐FLAG M2 Affinity Gel (beads) (50 μl/sample) was washed twice with 1 ml of 1× RIPA buffer. Finally, the prepared lysate supernatant was transferred to a 1.5‐ml tube containing the EZView Red Anti‐FLAG M2 Affinity Gel (beads) after two washes in 1 ml of 1× RIPA buffer and incubated at 4°C overnight with rotation.

#### Immunoprecipitation

The beads were harvested by centrifugation (1500 rpm for 1 min at 4°C) and washed three times with 1.5 ml of 1× RIPA buffer containing 1 mM PMSF by rotation for 5 min at 4°C and spinning for 30 s. Using the same methods, the beads were washed three times in 1.5 ml of LiCl/detergent solution (10 mM Tris‐HCl pH 8.0, 250 mM LiCl, 1 mM EDTA pH 8.0, 0.5% NP‐40, 0.5% sodium deoxycholate) and three times in 1.5 ml of Tris‐EDTA (TE) (pH 8.0). After removing the supernatant, 300 μl of 0.1 M glycine‐HCl (pH 3.5) was added to the beads, followed by incubation with rotation for 10 min at RT to elute the RNA–protein complex. The supernatant was then collected by centrifugation at 1500 rpm for 1 min and transferred to a 0.5‐ml tube containing 30 μl of elution buffer.

#### RNA purification

The RNA–protein complex was treated with RNase A (7000 units/ml) at 37°C for 1 h, and then incubated at 65°C for 8 h to reverse the cross‐linking. RNA was then immediately isolated using TRIzol reagent (Transgen) following the manufacturer's instructions.

### Reverse transcription

cDNA was synthesized using 300 ng of RNA as a template in a 10‐μl reaction mixture using the PrimeScript first strand cDNA Synthesis Kit (TaKaRa) as described in the manufacturer's protocol, and the cDNA product was detected by electrophoresis.

### Real‐time relative qPCR

Cells of the MOR1378 (wt) strain and those of the *dnaA*_A345S_, wt/control vector, and wt/p*dnaA*116 (DnaA overproducer) strains were exponentially grown in ABTGcasa medium at 37°C. cDNA was synthesized as described above. The HisL and HisG RNA levels were measured by RT‐qPCR using the appropriate primer pairs (Table S3) with the cDNA as a template. The primers for qPCR were designed to have Tm values of 55–60°C and amplify products shorter than 150 bp. qPCR was performed using the SYBR Premix ExTaq II kit (TliRNaseH Plus) (TaKaRa) in a Roche 488 II Real‐Time PCR System (Roche) using default cycling conditions (an initial incubation at 95°C for 30 s, followed by 45 cycles of 95°C for 5 s and 60°C for 20 s). Melting curve analysis was performed as described previously^54^. The relative expression of the target genes was calculated using the $2^{‐∆∆C_{t}}$ method.

### Northern blot analysis

Total RNA was extracted from wild‐type (wt) MOR1378 and derived (*dnaA*_A345S_, wt/control vector, and wt/p*dnaA*116 [DnaA overproducer] cells) using TRIzol reagent (Transgene) according to the manufacturer's protocol. The *hisG* gene was amplified using the pTAC3953‐hisG‐F and pTAC3953‐hisG‐R primer pair and subsequently inserted into the pTAC3953 plasmid for digoxin labeling. Single‐stranded DNA of the *hisL* gene (51 bp) was directly synthesized by Sagan Corporation, and also for digoxin labeling. The *hisG* and *hisL* fragments were labeled with digoxin using the northern blot probe stock solution following the protocol of the DIG High Prime DNA Labeling and Detection Starter Kit I (Roche). Total RNA (1 μg) from each strain was denatured in agarose gel‐formaldehyde, transferred to a Hybond‐N+ membrane (Solarbio) by capillary transfer, and cross‐linked with UV‐light. The membrane was then incubated with the digoxin‐labeled *hisG* or *hisL* probe. The appropriate hybridization temperature (Tm [HisL] = 30°C and Tm [HisG] = 65°C) was obtained by calculating the GC content and percent homology of the probe to target RNA. The membrane was developed using NBT/BCIP after washed and then visualized using the ChemiDoc XRS system (Bio‐Rad) in Image Lab Version 5.2.1 (Bio‐Rad). Stripping and reprobing of the blots were performed according to the manufacturer's protocol to detect HisL and HisG independently.

### Measurement of the fitness of Δ*hisL* and Δ*hisL‐SL* mutant cells

Wt MOR1378 cells and the derived Δ*hisL* (MOR2062) and Δ*hisL‐SL* (MOR2063) cells (Table S1) were exponentially grown in ABTGcasa medium at 37°C. A total of 100 μl of culture from each bacterial strain was taken at OD_450_ = 0.4 (10^0^ cell dilution) and diluted to 10^−1^ by mixing 10 μl of the 10^0^ cell dilution with 90 μl of ABTGcasa medium. Similarly, 10^−2^, 10^−3^, 10^−4^, 10^−5^, and 10^−6^ dilutions were prepared. Mixtures of wt and Δ*hisL* or Δ*hisL‐SL* cells were obtained using the dilutions mentioned above^55^, ^56^. Δ*hisL*/wt mixtures containing 46% and 88% Δ*hisL* cells were prepared by, respectively, mixing 10 and 90 μl of Δ*hisL* mutant cells with 10 μl of wt cells from the 10^−5^ dilutions. Using similar quantities and dilutions, Δ*hisL‐SL*/wt mixtures containing, respectively, 53% and 91% Δ*hisL‐SL* cells were prepared. The cell mixtures were grown in ABTGcasa medium at 37°C and diluted in fresh medium every 8–12 h. On days 4 and 7, each cell mixture was plated on LB agar in dilutions of 10^−2^, 10^−3^, 10^−4^, 10^−5^, and 10^−6^ overnight at 37°C and the number of well‐separated colonies on each plate was counted the next day. The number of cells in each sample (10 μl) was calculated according to the following formula^57^:

$$N=\Sigma c/\left(n_{1}+0.1n_{2}\right)d$$

where *N* is the number of cells in a 10 μl sample; Σc is the sum of colonies from plates for two consecutive dilutions, where at least one of the plates contains more than 15 colonies; $n_{1}$ is the number of plates for the first selected dilution; $n_{2}$ is the number of plates for the second selected dilution; and *d* is the dilution factor corresponding to the first dilution.

The chromosomal *hisL* and *hisL‐SL* regions in each colony were detected by PCR using the Fw‐hisLp and Rev‐hisL‐SL primers to identify Δ*hisL* and Δ*hisL‐SL* cells.

### Statistical analysis

All statistical analyses were performed using GraphPad Prism 9.0 for Mac. Descriptive statistics, one‐way ANOVA, one‐sample *t* tests, and paired samples *t* tests were used.

## AUTHOR CONTRIBUTIONS

**Yuan Yao**: Conceptualization (equal); data curation (lead); formal analysis (lead); methodology (equal); writing—original draft (equal); writing—review and editing (equal). **Hongwei Sun**: Methodology (equal); writing—original draft (equal). **Wurihan**: Investigation (equal). **Gegeheng**: Methodology (equal). **Gezi**: Methodology (equal). **Kirsten Skarstad**: Writing—original draft (equal); writing—review and editing (equal). **Lifei Fan**: Conceptualization (equal); resources (equal); writing—original draft (equal). **Morigen**: Conceptualization (lead); project administration (lead); resources (lead); supervision (lead); writing—original draft (lead); writing—review and editing (lead).

## ETHICS STATEMENT

This article does not contain any studies with human participants or animals performed by any of the authors.

## CONFLICT OF INTERESTS

The authors declare no conflict of interests.

## Supporting information

Supporting information.

## Data Availability

All data used during the study appear in the submitted article.

## References

[1] Keller EB , Calvo JM . Alternative secondary structures of leader RNAs and the regulation of the *trp*, *phe*, *his*, *thr*, and *leu* operons. Proc Natl Acad Sci USA. 1979;76:6186–90. 392514 10.1073/pnas.76.12.6186 PMC411828

[2] Kolter R , Yanofsky C . Attenuation in amino acid biosynthetic operons. Annu Rev Genet. 1982;16:113–34. 6186194 10.1146/annurev.ge.16.120182.000553

[3] Lee F , Yanofsky C . Transcription termination at the *trp* operon attenuators of *Escherichia coli* and *Salmonella typhimurium*: RNA secondary structure and regulation of termination. Proc Natl Acad Sci USA. 1977;74:4365–9. 337297 10.1073/pnas.74.10.4365 PMC431942

[4] Johnston HM , Barnes WM , Chumley FG , Bossi L , Roth JR . Model for regulation of the histidine operon of *Salmonella* . Proc Natl Acad Sci USA. 1980;77:508–12. 6987654 10.1073/pnas.77.1.508 PMC348301

[5] Lawrence JG . Shared strategies in gene organization among prokaryotes and eukaryotes. Cell. 2002;110:407–13. 12202031 10.1016/s0092-8674(02)00900-5

[6] Artz SW , Broach JR . Histidine regulation in *Salmonella typhimurium*: an activator attenuator model of gene regulation. Proc Natl Acad Sci USA. 1975;72:3453–7. 1103149 10.1073/pnas.72.9.3453 PMC433012

[7] Kasai T . Regulation of the expression of the histidine operon in *Salmonella typhimurium* . Nature. 1974;249:523–7. 4599761 10.1038/249523a0

[8] Bertrand K , Korn L , Lee F , Platt T , Squires CL , Squires C , et al. New features of the regulation of the tryptophan operon. Science. 1975;189:22–6. 1094538 10.1126/science.1094538

[9] Zurawski G , Elseviers D , Stauffer GV , Yanofsky C . Translational control of transcription termination at the attenuator of the *Escherichia coli* tryptophan operon. Proc Natl Acad Sci USA. 1978;75:5988–92. 366606 10.1073/pnas.75.12.5988 PMC393102

[10] Zurawski G , Brown K , Killingly D , Yanofsky C . Nucleotide sequence of the leader region of the phenylalanine operon of *Escherichia coli* . Proc Natl Acad Sci USA. 1978;75:4271–5. 360214 10.1073/pnas.75.9.4271 PMC336095

[11] Gardner JF . Regulation of the threonine operon: tandem threonine and isoleucine codons in the control region and translational control of transcription termination. Proc Natl Acad Sci USA. 1979;76:1706–10. 287010 10.1073/pnas.76.4.1706 PMC383459

[12] Gemmill RM , Wessler SR , Keller EB , Calvo JM . *leu* operon of *Salmonella typhimurium* is controlled by an attenuation mechanism. Proc Natl Acad Sci USA. 1979;76:4941–5. 388423 10.1073/pnas.76.10.4941 PMC413054

[13] Barnes WM . DNA sequence from the histidine operon control region: seven histidine codons in a row. Proc Natl Acad Sci USA. 1978;75:4281–5. 360216 10.1073/pnas.75.9.4281 PMC336097

[14] Di Nocera PP , Blasi F , Di Lauro R , Frunzio R , Bruni CB . Nucleotide sequence of the attenuator region of the histidine operon of *Escherichia coli* K‐12. Proc Natl Acad Sci USA. 1978;75:4276–80. 360215 10.1073/pnas.75.9.4276 PMC336096

[15] Artz S , Holzschu D , Blum P , Shand R . Use of M13mp phages to study gene regulation, structure and function: cloning and recombinational analysis of genes of the *Salmonella typhimurium* histidine operon. Gene. 1983;26:147–58. 6323256 10.1016/0378-1119(83)90184-1

[16] Winkler ME , Roth DJ , Hartman PE . Promoter‐ and attenuator‐related metabolic regulation of the *Salmonella typhimurium* histidine operon. J Bacteriol. 1978;133:830–43. 342509 10.1128/jb.133.2.830-843.1978 PMC222095

[17] Bachmann BJ . Linkage map of *Escherichia coli* K12, edition 7. Microbiol Rev. 1983;47:180–230. 6348505 10.1128/mr.47.2.180-230.1983 PMC281571

[18] Verde P , Frunzio R , Paolo di Nocera P , Blasi F , Bruni CB . Identification, nucleotide sequence and expression of the regulatory region of the histidine operon of *Escherichia coli* K‐12. Nucleic Acids Res. 1981;9:2075–86. 6170941 10.1093/nar/9.9.2075 PMC326827

[19] Frunzio R , Bruni CB , Blasi F . In vivo and in vitro detection of the leader RNA of the histidine operon of *Escherichia coli* K‐12. Proc Natl Acad Sci USA. 1981;78:2767–71. 6166940 10.1073/pnas.78.5.2767 PMC319438

[20] Lewis JA , Ames BN . Histidine regulation in *Salmonella typhimurium* . J Mol Biol. 1972;66:131–42. 4339187 10.1016/s0022-2836(72)80011-1

[21] Riggs DL , Mueller RD , Kwan HS , Artz SW . Promoter domain mediates guanosine tetraphosphate activation of the histidine operon. Proc Natl Acad Sci USA. 1986;83:9333–7. 3540936 10.1073/pnas.83.24.9333 PMC387132

[22] Da Costa XJ , Artz SW . Mutations that render the promoter of the histidine operon of *Salmonella typhimurium* insensitive to nutrient‐rich medium repression and amino acid downshift. J Bacteriol. 1997;179:5211–7. 9260966 10.1128/jb.179.16.5211-5217.1997 PMC179382

[23] Ames BN , Tsang TH , Buck M , Christman MF . The leader mRNA of the histidine attenuator region resembles tRNA^His^: possible general regulatory implications. Proc Natl Acad Sci USA. 1983;80:5240–2. 6351055 10.1073/pnas.80.17.5240 PMC384228

[24] Rudd KE , Menzel R . *his* operons of *Escherichia coli* and *Salmonella typhimurium* are regulated by DNA supercoiling. Proc Natl Acad Sci USA. 1987;84:517–21. 3025879 10.1073/pnas.84.2.517 PMC304240

[25] Fuller RS , Funnell BE , Kornberg A . The dnaA protein complex with the *E. coli* chromosomal replication origin (*oriC*) and other DNA sites. Cell. 1984;38:889–900. 6091903 10.1016/0092-8674(84)90284-8

[26] Messer W , Weigel C . DnaA initiator–also a transcription factor. Mol Microbiol. 1997;24:1–6. 9140960 10.1046/j.1365-2958.1997.3171678.x

[27] Blancpotard AB , Figueroabossi N , Bossi L . Histidine operon deattenuation in *dnaA* mutants of *Salmonella typhimurium* correlates with a decrease in the gene dosage ratio between tRNA^His^ and histidine biosynthetic loci. J Bacteriol. 1999;181:2938–41. 10217789 10.1128/jb.181.9.2938-2941.1999 PMC93740

[28] Mironov AS , Gusarov I , Rafikov R , Lopez LE , Shatalin K , Kreneva RA , et al. Sensing small molecules by nascent RNA. Cell. 2002;111:747–56. 12464185 10.1016/s0092-8674(02)01134-0

[29] Nahvi A , Sudarsan N , Ebert MS , Zou X , Brown KL , Breaker RR . Genetic control by a metabolite binding mRNA. Chem Biol. 2002;9:1043–9. 12323379 10.1016/s1074-5521(02)00224-7

[30] Winkler W , Nahvi A , Breaker RR . Thiamine derivatives bind messenger RNAs directly to regulate bacterial gene expression. Nature. 2002;419:952–6. 12410317 10.1038/nature01145

[31] Winkler WC , Nahvi A , Sudarsan N , Barrick JE , Breaker RR . An mRNA structure that controls gene expression by binding S‐adenosylmethionine. Nat Struct Mol Biol. 2003;10:701–7. 10.1038/nsb967 12910260

[32] McCown PJ , Corbino KA , Stav S , Sherlock ME , Breaker RR . Riboswitch diversity and distribution. RNA. 2017;23:995–1011. 28396576 10.1261/rna.061234.117 PMC5473149

[33] Pavlova N , Kaloudas D , Penchovsky R . Riboswitch distribution, structure, and function in bacteria. Gene. 2019;708:38–48. 31128223 10.1016/j.gene.2019.05.036

[34] Venkata Subbaiah KC , Hedaya O , Wu J , Jiang F , Yao P . Mammalian RNA switches: molecular rheostats in gene regulation, disease, and medicine. Comput Struct Biotechnol J. 2019;17:1326–38. 31741723 10.1016/j.csbj.2019.10.001 PMC6849081

[35] Serganov A , Nudler E . A decade of Riboswitches. Cell. 2013;152:17–24. 23332744 10.1016/j.cell.2012.12.024 PMC4215550

[36] Cromie MJ , Shi Y , Latifi T , Groisman EA . An RNA sensor for intracellular Mg^2+^ . Cell. 2006;125:71–84. 16615891 10.1016/j.cell.2006.01.043

[37] Hollands K , Proshkin S , Sklyarova S , Epshtein V , Mironov A , Nudler E , et al. Riboswitch control of Rho‐dependent transcription termination. Proc Natl Acad Sci USA. 2012;109:5376–81. 22431636 10.1073/pnas.1112211109 PMC3325659

[38] Miller RV , Ripp S , Replicon J , Ogunseitan O , Kokjohn TA . Virus‐mediated gene transfer in freshwater environments. In: Gene transfers and environment. New York, NY, USA: Springer; 1992. p. 51–62.

[39] Morigen M , Boye E , Skarstad K , Løbner‐Olesen A . Regulation of chromosomal replication by DnaA protein availability in *Escherichia coli*: effects of the datA region. Biochim Biophys Acta. 2001;1521:73–80. 11690638 10.1016/s0167-4781(01)00292-5

[40] Zuker M . Mfold web server for nucleic acid folding and hybridization prediction. Nucleic Acids Res. 2003;31:3406–15. 12824337 10.1093/nar/gkg595 PMC169194

[41] Wurihan , Gezi , Brambilla E , Wang S , Sun H , Fan L , et al. DnaA and LexA proteins regulate transcription of the *uvrB* gene in *Escherichia coli*: the role of DnaA in the control of the SOS regulon. Front Microbiol. 2018;9:1212. 29967594 10.3389/fmicb.2018.01212 PMC6015884

[42] Krause M , Rückert B , Lurz R , Messer W . Complexes at the replication origin of *Bacillus subtilis* with homologous and heterologous DnaA protein. J Mol Biol. 1997;274:365–80. 9405146 10.1006/jmbi.1997.1404

[43] Ferenci T , Zhou Z , Betteridge T , Ren Y , Liu Y , Feng L , et al. Genomic sequencing reveals regulatory mutations and recombinational events in the widely used MC4100 lineage of *Escherichia coli* K‐12. J Bacteriol. 2009;191:4025–9. 19376874 10.1128/JB.00118-09 PMC2698400

[44] Casadaban MJ . Transposition and fusion of the *lac* genes to selected promoters in *Escherichia coli* using bacteriophage lambda and Mu. J Mol Biol. 1976;104:541–55. 781293 10.1016/0022-2836(76)90119-4

[45] Hansen EB , Atlung T , Hansen FG , Skovgaard O , von Mevenburg K . Fine structure genetic map and complementation analysis of mutations in the *dnaA* gene of *Escherichia coli* . Mol Gen Genet. 1984;196:387–96. 6094968 10.1007/BF00436184

[46] Selth LA , Gilbert C , Svejstrup JQ . RNA immunoprecipitation to determine RNA‐protein associations in vivo. Cold Spring Harbor Protocols. 2009;2009:pdb.prot5234. 20147192 10.1101/pdb.prot5234

[47] Zheng L . An efficient one‐step site‐directed and site‐saturation mutagenesis protocol. Nucleic Acids Res. 2004;32:e115. 15304544 10.1093/nar/gnh110 PMC514394

[48] Kovach JS , Phang JM , Blasi F , Barton RW , Ballesterosolmo A , Goldberger RF . Interaction between histidyl transfer ribonucleic acid and the first enzyme for histidine biosynthesis of *Salmonella typhimurium* . J Bacteriol. 1970;104:787–92. 4923072 10.1128/jb.104.2.787-792.1970 PMC285059

[49] Datsenko KA , Wanner BL . One‐step inactivation of chromosomal genes in *Escherichia coli* K‐12 using PCR products. Proc Natl Acad Sci USA. 2000;97:6640–5. 10829079 10.1073/pnas.120163297 PMC18686

[50] Ellermeier CD , Janakiraman A , Slauch JM . Construction of targeted single copy lac fusions using λ Red and FLP‐mediated site‐specific recombination in bacteria. Gene. 2002;290:153–61. 12062810 10.1016/s0378-1119(02)00551-6

[51] Lennox ES . Transduction of linked genetic characters of the host by bacteriophage P1. Virology. 1955;1:190–206. 13267987 10.1016/0042-6822(55)90016-7

[52] Morigen N , Molina F , Skarstad K . Deletion of the *datA* site does not affect once‐per‐cell‐cycle timing but induces rifampin‐resistant replication. J Bacteriol. 2005;187:3913–20. 15939703 10.1128/JB.187.12.3913-3920.2005 PMC1151742

[53] Brøndsted L , Atlung T . Anaerobic regulation of the hydrogenase 1 (*hya*) operon of *Escherichia coli* . J Bacteriol. 1994;176:5423–8. 8071220 10.1128/jb.176.17.5423-5428.1994 PMC196730

[54] Yao Y , Fan L , Shi Y , Odsbu I . Morigen. A spatial control for correct timing of gene expression during the *Escherichia coli* cell cycle. Genes. 2016;8:1. 28025549 10.3390/genes8010001 PMC5294996

[55] Elena SF , Ekunwe L , Hajela N , Oden SA , Lenski RE . Distribution of fitness effects caused by random insertion mutations in *Escherichia coli* . Genetica. 1998;102/103:349–58. 9720287

[56] Remold SK , Lenski RE . Pervasive joint influence of epistasis and plasticity on mutational effects in *Escherichia coli* . Nat Genet. 2004;36:423–6. 15072075 10.1038/ng1324

[57] Landfeld A , Strohalm J , Kýhos K , Průchová J , Houška M , Novotná P , et al. High‐pressure inactivation of *Enterococcus faecium*—modelling and verification. Czech J Food Sci. 2009;27:134–41.

