# Supplementary material for: A DnaA‐dependent riboswitch for transcription attenuation of the his operon
Source: mLife. 2023 Jun 30;2(2):126–40. doi: 10.1002/mlf2.12075 (PMC10989985; doi:10.1002/mlf2.12075)
Supplement: Supplementary file 1 — Supporting information. [file MLF2-2-126-s001.docx]

Supporting Information for

**A DnaA-dependent riboswitch for transcription attenuation of the *his* operon**

Yuan Yao^1,†^, Hongwei Sun^1,†^, Wurihan^1,†^, Gegeheng^1,†^, Gezi^1^, Kirsten Skarstad^2,^*, Lifei Fan^1,^*, Morigen^1,^*

*Corresponding authors:

Kirsten Skarstad

Email: kirsten.skarstad@rr-research.no

Lifei Fan

Email: lifei.fan@imu.edu.cn

Morigen

Email: morigenm@hotmail.com

SI Figures


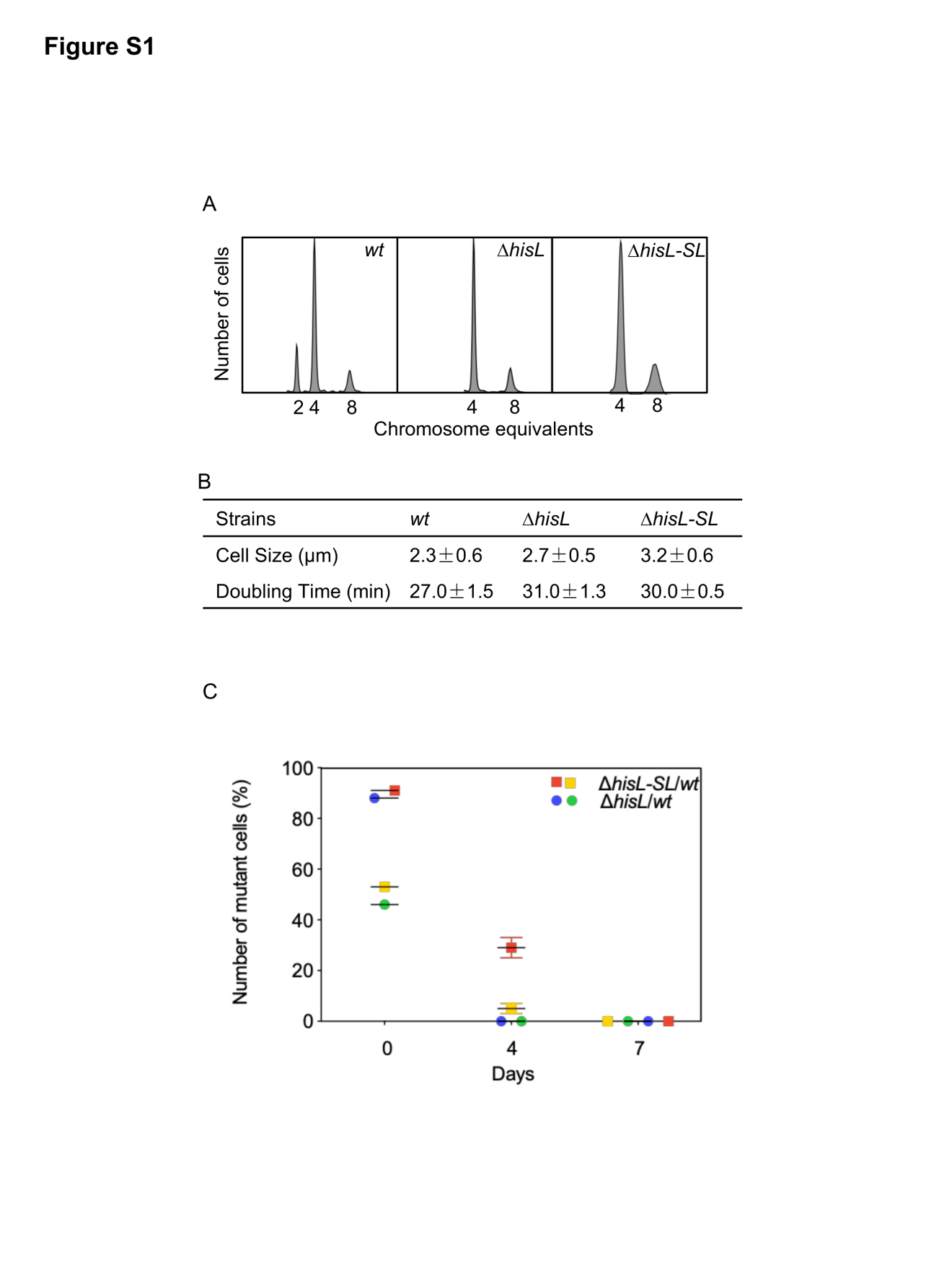


**Fig. S1. Deletion of the *hisL* or *hisL-SL* region leads to premature initiation of replication.** Cells of the wild-type (wt) MOR1378 strain and those of its ∆*hisL* and ∆*hisL-SL* derivatives were exponentially grown in ABTGcasa medium at 37 °C and treated with rifampicin and cephalexin for 3–4 generations. Subsequently, the cells were fixed in 70% ethanol and analyzed by flow cytometry (BD LSRFortessa) after staining with Hoechst 33258, as previously described ^1^. A total of 10,000 cells were assessed for each analysis (**A**). Cell size (μm) and doubling time (min) were determined as previously described ^1^ (**B**). **The *hisL* and *hisL-SL* regions bestow increased cell fitness** (**C**). Cells of the wt MOR1378 strain and those of its Δ*hisL* and Δ*hisL-SL* derivatives were exponentially grown in ABTGcasa medium at 37 °C. Cell fitness was measured as described in Materials and Methods. Filled squares represent the proportion of Δ*hisL-SL* cells in the Δ*hisL-SL*/wt mixture (Red or yellow block), while filled circles indicate the proportion of Δ*hisL* cells in the Δ*hisL*/wt mixture (Blue or green round dot). Colors indicate the different initial proportions of mutant cells in the four mixtures. Error bars are as shown.


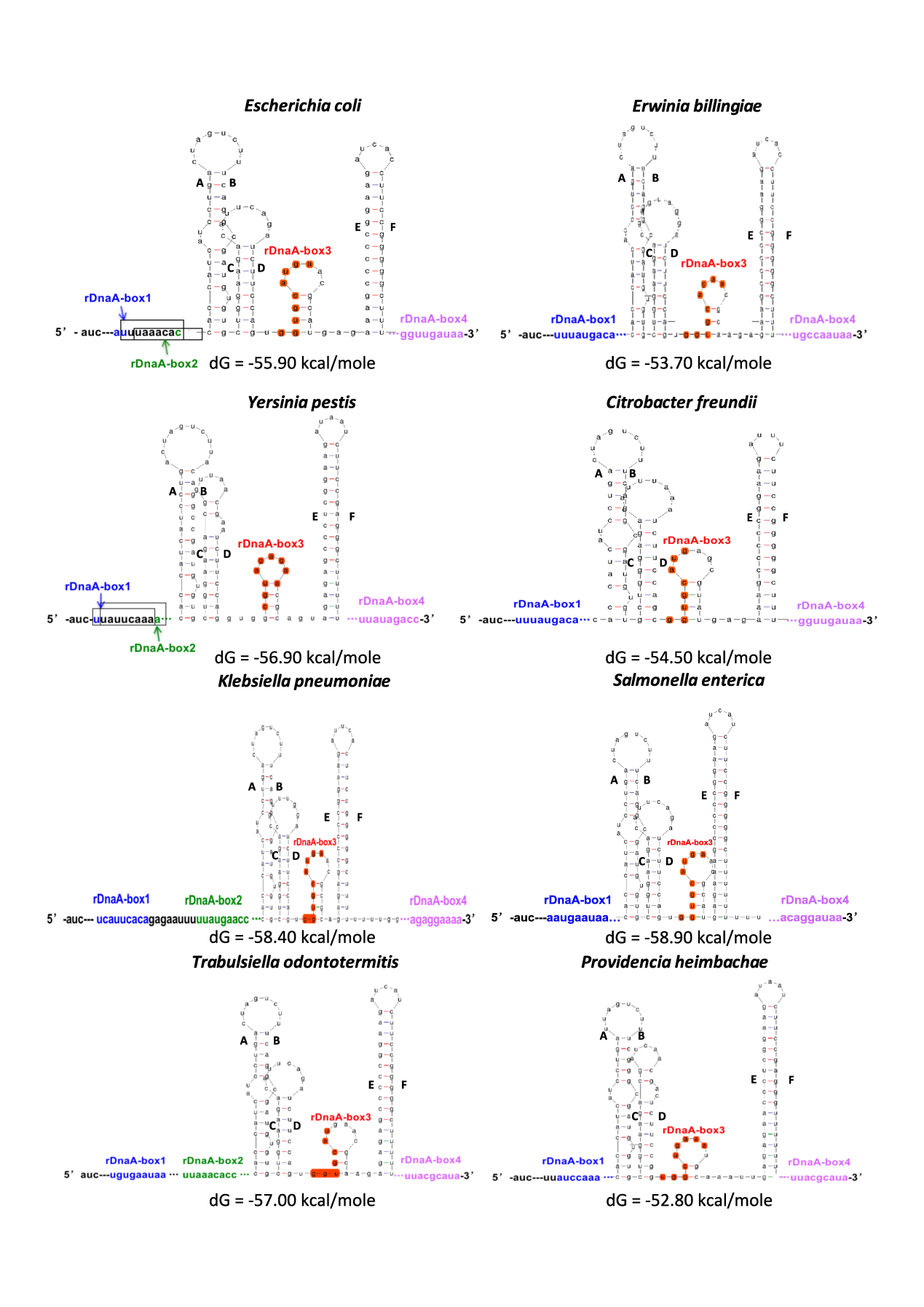


**Fig. S2. The predicted secondary structures of the HisL-SL RNA are conserved in bacteria.** Schematic representations of the secondary structures of the HisL-SL (stem-loop) RNA (112 nucleotides) from *Escherichia coli*, *Erwinia billingiae*, *Yersinia* *pestis*, *Citrobacter* *freundii*, *Klebsiella* *pneumoniae*, *Salmonella* *enterica*, *Trabulsiella* *odontotermitis*, and *Providencia* *heimbachae* as predicted by the Mfold program. Sequences in color represent rDnaA boxes. The folding energy is presented below the schematic representation.


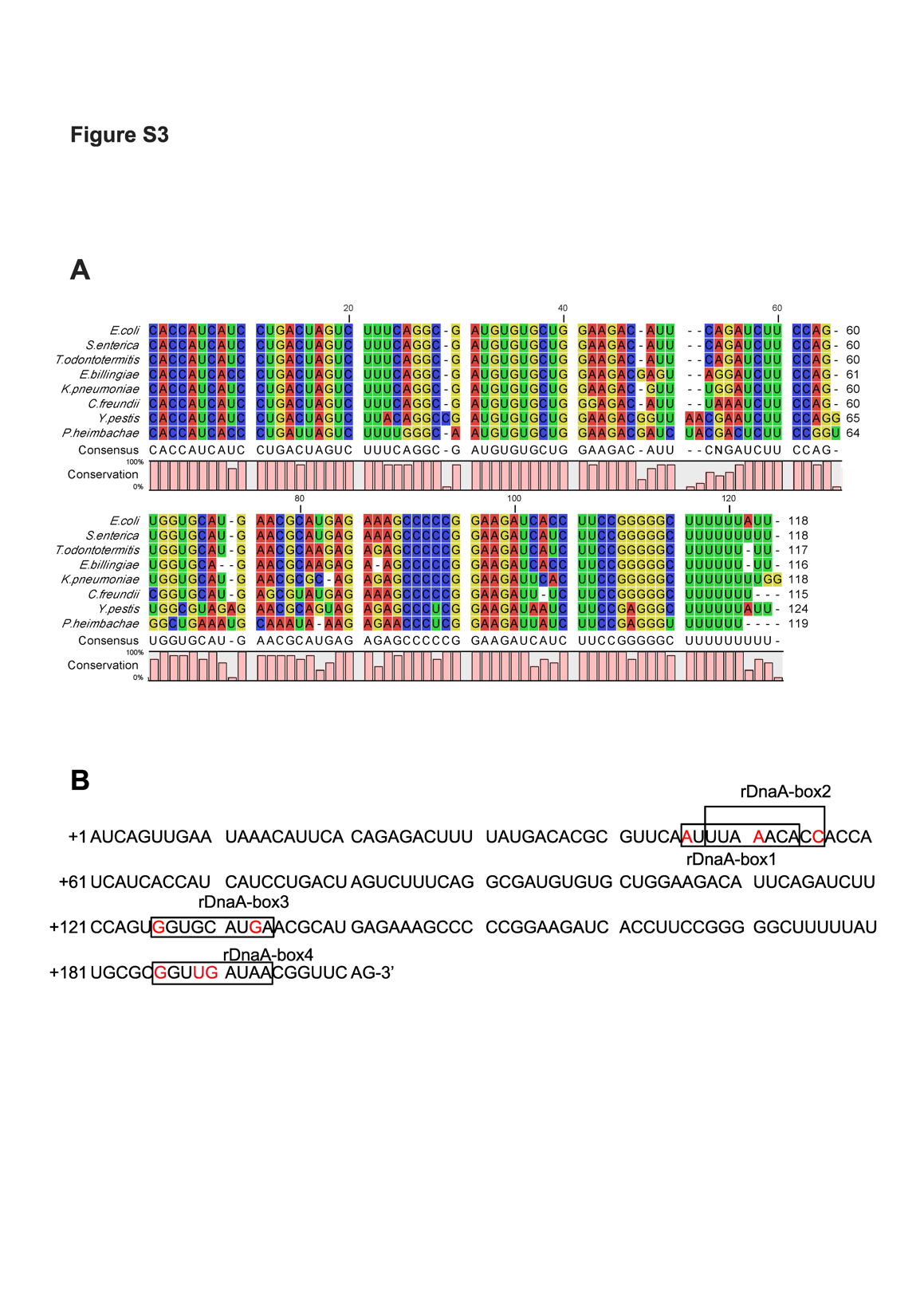


**Fig. S3. The HisL-SL RNA sequence is conserved in Gram-negative bacteria.** (A) The HisL-SL RNA sequences from nine bacteria were aligned using CLC Sequence Viewer 7.0 software. The names of the bacteria are indicated on the left. Regions in purple represent highly conserved sequences in all the bacteria analyzed; those in pink indicate conserved sequences with few variations; and those in turquoise represent sequences with several variations. (B) The HisL-SL RNA contains four rDnaA boxes. rDnaA boxes are as boxed and indicated. rDnaA boxes 1 and 2 overlap over 7 nucleotides (position 46–56 upstream of the transcriptional start site [TSS]). rDnaA box 3 is located at position 126–134 and rDnaA box 4 at position 187–195 downstream of the TSS. Mismatches compared with the consensus sequence of the rDnaA box (UUA/UUNCACA) are in red.


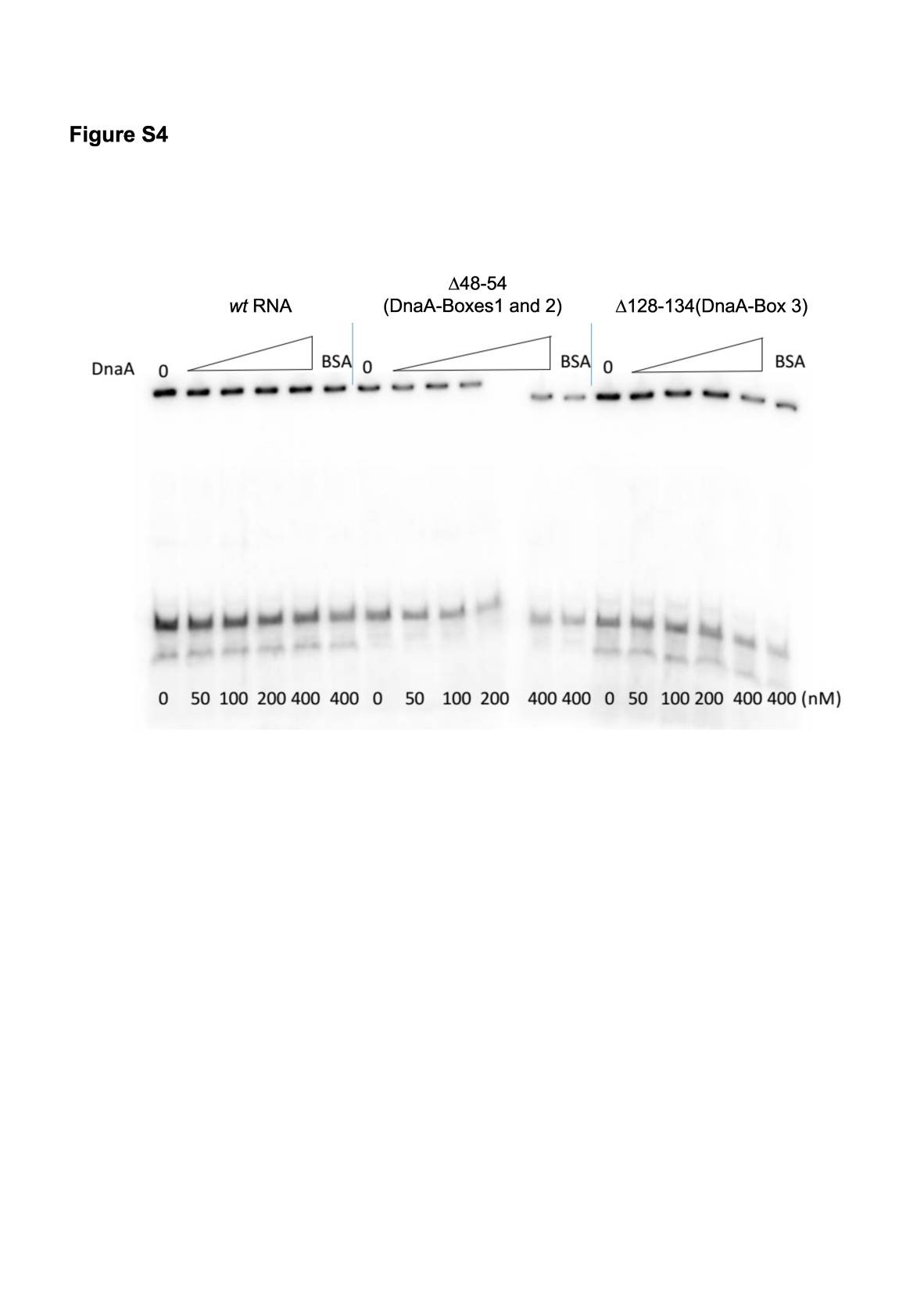


**Fig. S4. DnaA does not interact with the HisL-SL RNA *in vitro*.** The HisL-SL (wt), HisL-SL (∆44–54), and HisL-SL (∆128–136) RNA fragments (100 ng) were labeled with α-^32^P-UTP and incubated with increasing amounts of ATP-DnaA or BSA at 37 °C for 20 min. The mixtures were subjected to 5% acrylamide gel electrophoresis. The dried gel was exposed for 2 h and autoradiographed. The amounts of ATP-DnaA and BSA are as indicated at the bottom of the gel and the RNA bands are as shown.


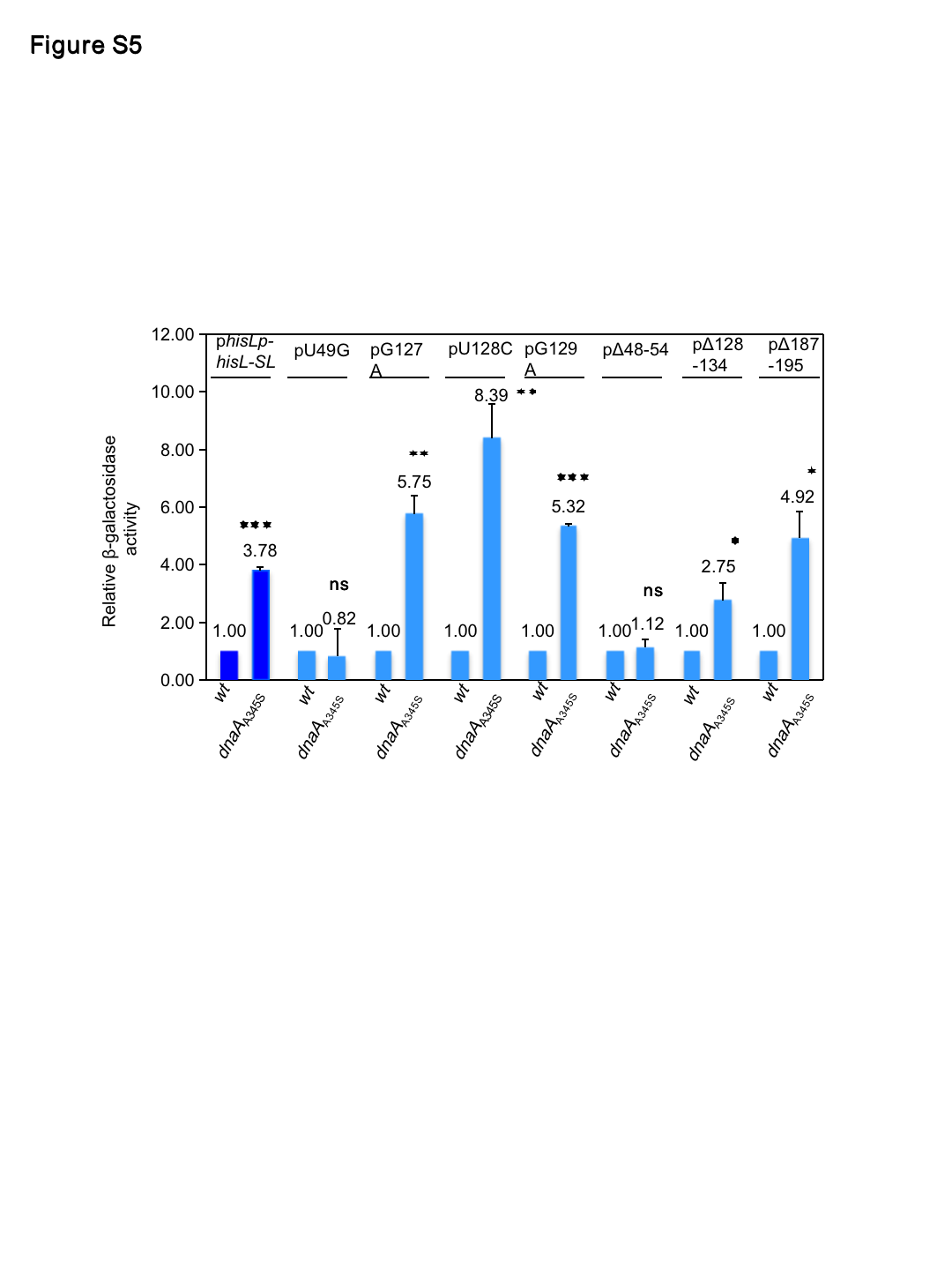


**Fig. S5. *his* transcription is not increased in the *dnaA*_A345S_ mutant when rDnaA box 1/2 is partially deleted.** β-Galactosidase activity in the p*hisLp*-*hisL*-*SL*, pU49G, pG127A, pU128C, pG129A, p∆48–54, p∆128–134, and p∆187–195 plasmids in MC4100 *dnaA*_A345S_ cells was measured relative to that of the same plasmids in wild-type MC4100 cells. Plasmids are as shown on top of the columns. Values are the averages of three individual experiments and the standard deviations are as given. ****P* < 0.001, ***P* < 0.01, **P* < 0.05, one-way analysis of variance (ANOVA).


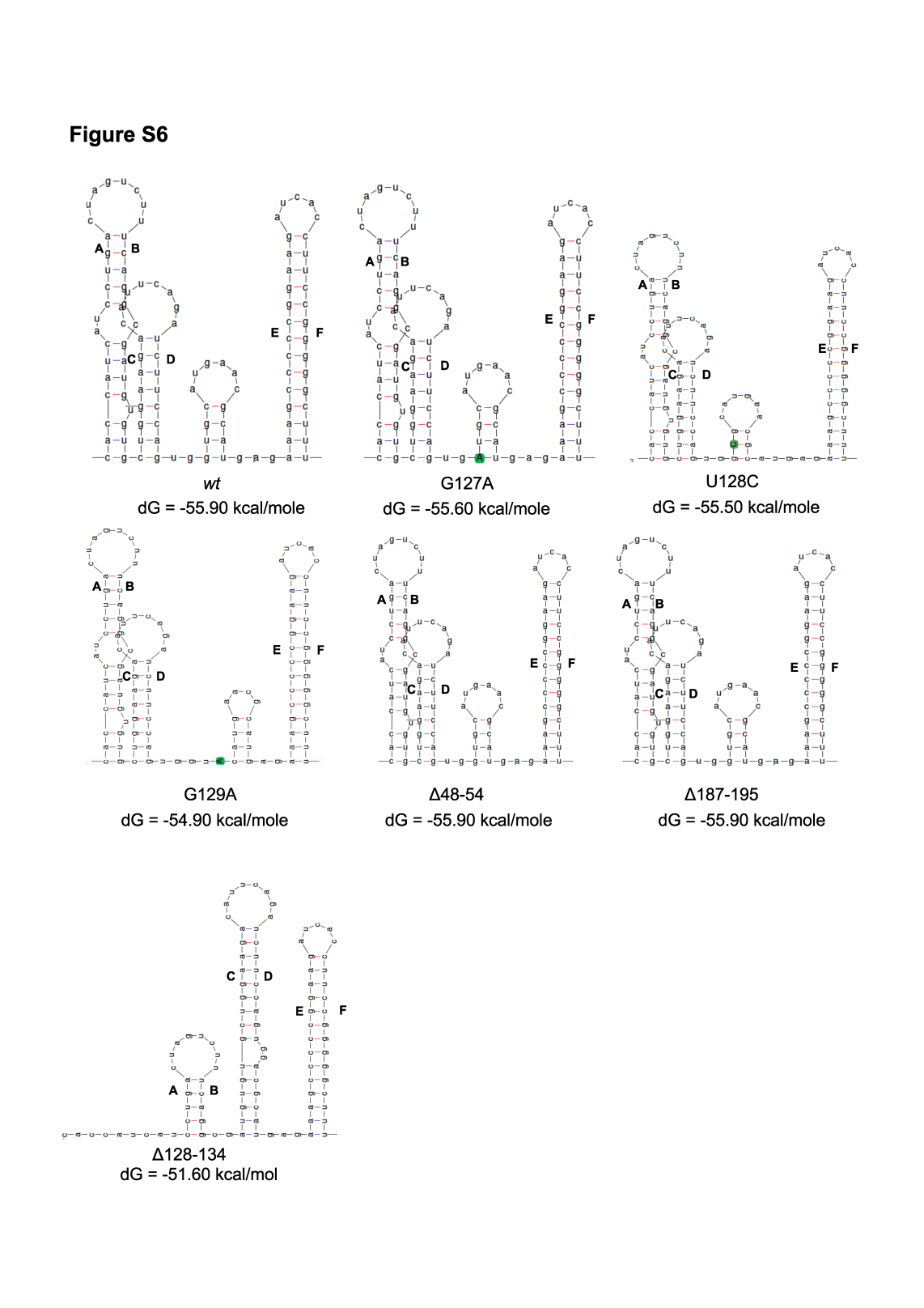


**Fig. S6. Mutations in the rDnaA boxes of the HisL-SL RNA do not affect the formation of the EF attenuator.** Schematic representation of the secondary structure of the HisL-SL (stem-loop) RNA from *E. coli* as predicted by Mfold. The predictions included mutated HisL-SL RNA molecules (G127A, U128C, G129A, ∆48–54, ∆128–134, and ∆187–195). The nucleotide in green represents a mutation and the folding energy is presented below the schematic representation.


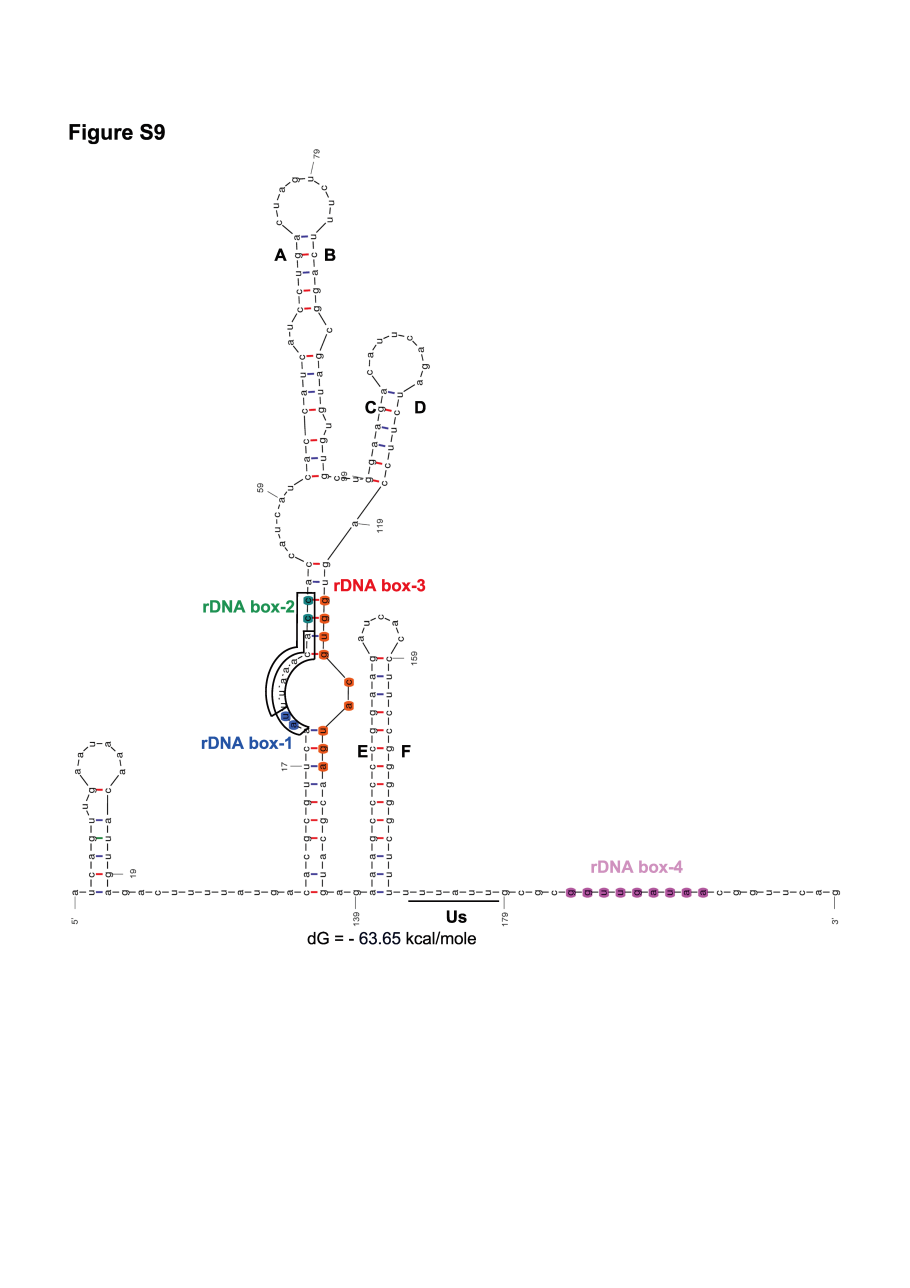


**Fig. S7. A double-stranded structure forms between rDnaA boxes 2 and 3 in the HisL-SL RNA.** Schematic representation of the secondary structure of the HisL-SL RNA (202 nucleotides) (described in Figure S3B) as predicted using Mfold. rDnaA boxes are shown in colors and the AB, CD, and EF stem-loops, followed by the uridines, are also shown. The folding energy is presented below the schematic representation.


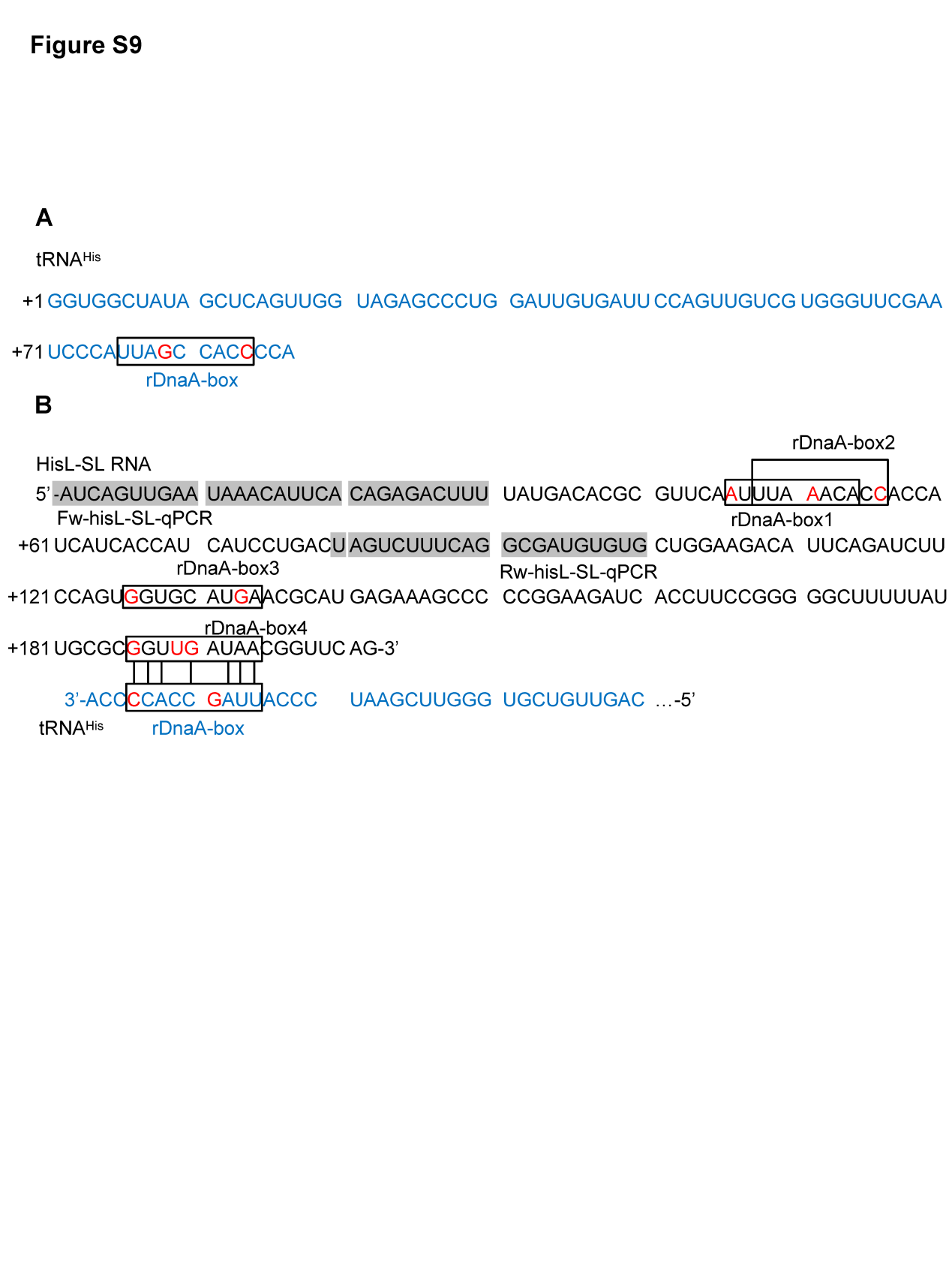


**Fig. S8. tRNA^His^ contains a rDnaA box through which it may base pair with the HisL-SL RNA.** A rDnaA box was identified in tRNA^His^; mismatched bases relative to the consensus sequence of the rDnaA box are in red (A). The rDnaA box in tRNA^His^ (in blue) was found to pair with rDnaA box 4 in the HisL-SL RNA (in black) (B)*.* rDnaA boxes are as boxed. The pair of primers indicated by gray background color was used to detect the *hisL-SL* region after PCR amplification of immunoprecipitated RNA.

Table S1. *E. coli* K12 strains used in this study.

| Strain | Genotype | Reference or source |
| --- | --- | --- |
| MC4100 | Wild type F^-^*araD*139 ∆(*lac*) U169 *strA thi* | ^2, 3^ |
| BL21-Gold (DE3) | B F^-^*ompT hsdS*B (r_B_^-^m_B_^-^) *dcm^+^* Tet^r^ *gal* (DE3) *endA* Hte | Agilent Technologies |
| SMG379 | MG1655 *dnaA*_A345S_…*cat* | ^4^ |
| CM735 | Wild type [*metE*46 *trp-*3 *his*-4 *thi*-1 *galK*2 *lacY*1 or *lacZ*4 *mtl*-1 *ara-*9 *tsx*-3 *ton*-1 *rpsL*8 or 9 *supE*44] *ilvE*12 λ^-b^ | ^5^ |
| MOR2082 | CM735 *dnaA*854*::Tn*l0(CM3452)*,lacZ::Tn*5 | This work |
| MOR749 | MC4100 *dnaA*_A345S_…*cat* | This work |
| MOR1378 | MC4100 *hisI*-*lacZ*…*neo* | This work |
| MOR1383 | MC4100 *hisI*-*lacZ*…*neo* *dnaA*_A345S_…*cat* | This work |
| MOR2062 | MC4100 Δ*hisL hisI*-*lacZ*…*neo* | This work |
| MOR2063 | MC4100 Δ*hisL-SL* *hisI*-*lacZ*…*neo* | This work |
| MOR2064 | MC4100 Δ*hisL* *dnaA*_A345S_…*cat* *hisI*-*lacZ*…*neo* | This work |
| MOR2065 | MC4100 Δ*hisL-SL* *dnaA*_A345S_…*cat hisI*-*lacZ*…*neo* | This work |
| WRH45 | MC4100 *dnaA*-*flag*…*cat* | This work |
| YBH0 | MC4100 Δ*hisL-SL dnaA*-*flag*…*cat hisI*-*lacZ*…*neo* | This work |

Table S2. Plasmids used in this study.

| Plasmid | Genotype | Reference or source |
| --- | --- | --- |
| pKD3 | *rep*_R6K_ *bla* FRT *cat* FRT | ^6^ |
| pKD4 | *rep*_R6K_ *bla* FRT *neo* FRT | ^6^ |
| pKD46 | *rep*_pSC101_^ts^*bla* P_araBAD_ γβ exo | ^6^ |
| pCP20 | *rep*_pSC101_^ts^ *bla cat* *cI*857P_R_ | ^6^ |
| pCE36 | *rep*_R6K_ *neo* FRT *lacZY* t*_his_* | ^7^ |
| pET-28a | *rep*_ColE1_ *neo lacI* P_T7_ | EMD Biosciences |
| p*dnaA*116 | *rep*_ColE1_ *bla lacI^+^*P_A1-03/04_ *dnaA* *ter_trpA_* | ^8^ |
| pLEX5BA | *rep*_ColE1_ *bla lacI^+^*P_A1-03/04_ *ter_trpA_* | ^8^ |
| pTAC3953 | *rep*_Pmd_ *neo lacZ* | ^9^ |
| pACYC177 | *rep*_p15A_*bla* *neo* | ^10^ |
| p*hisR* | The *hisR* gene with its native promoter was inserted onto pACYC177 at the *Bam*HI and *Hin*dIII sites. | This work |
| p*hisL*p-*hisL*-*SL* | The whole cluster of the *hisL* promoter, *hisL* gene and *SL* (stem-loop) region was inserted in front of the promoterless*lacZ* gene into pTAC3953 at the *Bam*HI and *Hin*dIII sites. | This work |
| p*hisL*p-*hisL* | A fragment of the *hisL* promoter and *hisL* gene was inserted onto pTAC3953 at the *Bam*HI and *Hin*dIII sites. | This work |
| p*hisL*p | The *hisL* promoter was inserted onto pTAC3953 at the *Bam*HI and *Hin*dIII sites as mentioned above. | This work |
| pU49G | T was replaced by G at the position of 49 in *hisL*-*SL* on p*hisL*p-*hisL*-*SL*. | This work |
| pG127A | G was replaced by A at the position of 127 in *hisL*-*SL* on p*hisL*p-*hisL*-*SL*. | This work |
| pU128C | T was replaced by C at the position of 128 in *hisL*-*SL* on p*hisL*p-*hisL*-*SL*. | This work |
| pG129A | G was replaced by A at the position of 129 in *hisL*-*SL* on p*hisL*p-*hisL*-*SL*. | This work |
| p∆48-54 | A fragment between 48 and 54 in *hisL*-*SL* was deleted on p*hisL*p-*hisL*-*SL*. | This work |
| p∆128-134 | A fragment between 128 and 134 in *hisL*-*SL* was deleted on p*hisL*p-*hisL-SL*. | This work |
| p∆187-195 | A fragment between 187 and 195 was deleted on p*hisL*p-*hisL-SL*. | This work |
| p*hisL*-TTG | A was replaced by T at the position of 32 in *hisL*-*SL* on p*hisL*p-*hisL*-*SL*. | This work |
| p*hisL*-TAA | TAA was inserted in front of the tandem *his* codons on p*hisL*p-*hisL-SL* | This work |
| p*hisL*-SD_mut_ | The SD site upstream of *hisL* was scrambled on p*hisL*p-*hisL*-*SL*(G23C)(G25C)(U28C)(U29C)(U30C)(U31C) | This work |

Table S3. Primers used in this study.

| Name of primer | Sequences (5' to 3') | Usage |
| --- | --- | --- |
| Fw-hisLp | CGCGGATCCCGTTCTGCGGGTATCTTTGA | To construct p*hisL*p with Rev-ds-hisLp; to construct p*hisL*p-*hisL* with Rev-hisL-gene; to construct p*hisL*p-*hisL*-*SL* with Rev-hisL-SL. |
| Rev-ds-hisLp | GCAAGCTTAGTCTCTGTGAATG | To construct p*hisL*p with Fw-hisLp as mentioned above |
| Rev-hisL-gene | CCCAAGCTTCTCATGCGTTCATGCACCACTG | To construct p*hisL*p-*hisL* with Fw-hisLp as mentioned above |
| Rev-hisL-SL | CCCAAGCTTCTGAACCGTTATCAACCGCGC | To construct p*hisL*p-*hisL*-*SL* with Fw-hisLp as mentioned above |
| Fw-hisL(50)-F-cat(20) | GTGGTTTAGGTTAAAAGACATCAGTTGAATAAACATTCACAGAGACTTTTGTGTAGGCTGGAGCTGCTTC | For deletion of *hisL* gene by one step inactivation on chromosome |
| Rev-ds-hisL(50)-F-cat(20) | ATGCACCACTGGAAGATCTGAATGTCTTCCAGCACACATCGCCTGAAAGAATGAATATCCTCCTTAG | For deletion of *hisL* gene by one step inactivation on chromosome |
| Fw-terminator(50)-F-cat(20) | ATGTGTGCTGGAAGACATTCAGATCTTCCAGTGGTGCATGAACGCATGAGGTGTAGGCTGGAGCTGCTTC | For deletion of *hisL-SL* region by one step inactivation on chromosome |
| Fw-terminator(50)-F-cat(20) | ATGTGTGCTGGAAGACATTCAGATCTTCCAGTGGTGCATGAACGCATGAGGTGTAGGCTGGAGCTGCTTC | For deletion of *hisL-SL* region by one step inactivation on chromosome |
| Fw-hisRp | CGCGGATCCGGCATCATCTTTATGCTGCTGGC | To amplify *hisR* and its promoter with RW-hisRp |
| RW-hisRp | CCCAAGCTTTGGGGTGGCTAATGGGATTC | To amplify *hisR* and its promoter with Fw-hisRp |
| H-hisI-cat-sn | GGCTGGATTTAACGACGGTAATTGAGAACCTGCGTAAACGGCATCAGTGACATATGAATATCCTCCTTAG | To insert the *lacZ* gene to downstream of the chromosomal *hisI* gene |
| H-hisI-cat-asn | CGTTTTTTTGCCGGATGCGGCAACAATATCGCATCCGCTTACCCCGCAACGTGTAGGCTGGAGCTGCTTC | To insert the *lacZ* gene to downstream of the chromosomal *hisI* gene |
| Fw-pT49G | ATGACACGCGTTCAATTGAAACACCACCAT | To introduce T49G in *hisL-SL* on p*hisL*p-*hisL-SL* |
| Rev-pT49G | CAATTGAACGCGTGTCATAAAAGTCTCTGT | To introduce T49G in *hisL-SL* on p*hisL*p-*hisL-SL* |
| Fw-pG127A | ATTCAGATCTTCCAGTGATGCATGAACGCA | To introduce G127A in *hisL-SL* on p*hisL*p-*hisL-SL* |
| Rev- pG127A | TCACTGGAAGATCTGAATGTCTTCCAGCAC | To introduce G127A in *hisL-SL* on p*hisL*p-*hisL-SL* |
| Fw-pT128C | CATTCAGATCTTCCAGTGGCGCATGAACGCAT | To intruduce T128C in *hisL-SL* on p*hisL*p-*hisL-SL* |
| Rev-pT128C | GCCACTGGAAGATCTGAATGTCTTCCAGCA | To introduce T128C in *hisL-SL* on p*hisL*p-*hisL-SL* |
| Fw-pG129A | TCAGATCTTCCAGTGGTACATGAACGCATG | To introduce G129A in *hisL-SL* on p*hisL*p-*hisL-SL* |
| Rev-pG129A | TACCACTGGAAGATCTGAATGTCTTCCAGC | To introduce G129A in *hisL-SL* on p*hisL*p-*hisL-SL* |
| Fw-pΔ48-54 | CGGGGTACCCCACCATCATCACCATCATCCTGACTAG | To delete 48-54 in *hisL-SL* on p*hisL*p-*hisL-SL* |
| Rev-pΔ48-54 | CGGGGTACCATTGAACGCGTGTCATAAAAGTCTCTGT | To delete 48-54 in *hisL-SL* on p*hisL*p-*hisL-SL* |
| Fw-pΔ128-134 | CGGGGTACCACGCATGAGAAAGCCCCCGGAAGATCAC | To delete 128-134 in *hisL-SL* on p*hisL*p-*hisL-SL* |
| Rev-pΔ128-134 | CGGGGTACCCCACTGGAAGATCTGAATGTCTTCCAGCACA- | To delete 128-134 in *hisL-SL* on p*hisL*p-*hisL-SL* |
| Fw-pΔ187-195 | CGGGGTACCCGGTTCAGAAGCTTCGAAGGCCTAGATCCG | To delete 187-195 in *hisL-SL* on p*hisL*p-*hisL-SL* |
| Rev-pΔ187-195 | CGGGGTACCGCGCAATAAAAAAGCCCCCGGAAGGTGATCTTC | To delete 187-195 in *hisL-SL* on p*hisL*p-*hisL-SL* |
| Fw-pA32T | ATTCACAGAGACTTTTGTGACACGCGTTC | To mutate initiation codon in *hisL* on p*hisL*p-*hisL-SL* |
| Rev-pA32T | CAAAAGTCTCTGTGAATGTTTATTCAACT | To mutate initiation codon in *hisL* on p*hisL*p-*hisL-SL* |
| Fw-pTAA | CGCGTTCAATTTAAATAACACCACCATC | To introduce TAA in front of the tandem *his* codons on p*hisL*p-*hisL-SL* |
| Rev-pTAA | TAATTTAAATTGAACGCGACAGTATTTTCAG | To introduce TAA in front of the tandem *his* codons on p*hisL*p-*hisL-SL* |
| HisL-SDmut-F | ATTCACACACACCCCCATGACACGCGTTCAAT | To introduce (G23C)(G25C) mutations on p*hisL*p-*hisL-SL* |
| HisL-SDmut-R | TGGGGGTGTGTGTGAATGTTTATTCAACTGATGTCTTTT | To introduce (G23C)(G25C) mutations on p*hisL*p-*hisL-SL* |
| HisL-SD-4Umut-F | ATTCACAGAGACCCCCATGACACGCGTTCAATTTAAACA | To introduce (U28C)(U29C)(U30C)(U31C) mutations on p*hisL*p-*hisL-SL* |
| HisL-SD-4Umut-R | ATGGGGGTCTCTGTGAATGTTTATTCAACTGATG | To introduce (U28C)(U29C)(U30C)(U31C) mutations on p*hisL*p-*hisL-SL* |
| pTAC3953-hisG-F | CGCGGATCCATGACAGACAACACTCGTTTACGC | To insert *hisG* into pTAC3953 |
| pTAC3953-hisG-R | CCCAAGCTTTCACTCCATCATCTTCTCAATCGG | To insert *hisG* into pTAC3953 |
| Fw-hisL-SL-qPCR | CATCAGTTGAATAAACATTCACAGAGAC | To detect *hisL-SL* by RT-qPCR |
| Rw-hisL-SL-qPCR | CACACATCGCCTGAAAGACTA | To detect *hisL-SL* by RT-qPCR |
| Fw-hisR | GGATCCGGCATCATCTTTATGCTGGC | To amplify the *hisR* gene with its promoter |
| Rw-hisR | CCCAAGCTTTGGGGTGGCTAATGGGATTC | To amplify the *hisR* gene with its promoter |
| Fw-gapA | CTGGTGCGAAGAAAGTGGTTAT | To detect *gapA* by RT-qPCR |
| Rw-gapA | TGAGTAGCGGTAGTAGCGTGAA | To detect *gapA* by RT-qPCR |
| Fw-hisR-RT | CGCGGATCCGGCATCATCTTTATGCTGCTGGC | To detect *hisR* by RT-PCR |
| Rw-hisR-RT | CCCAAGCTTTGGGGTGGCTAATGGGATTC | To detect *hisR* by RT-PCR |
| Fw-hisG-RT | CGCGGATCCGGCATCATCTTTATGCTGCTGGC | To detect *hisG* by RT-qPCR |
| Rw-hisG-RT | CGCGGATCCGGCATCATCTTTATGCTGCTGGC | To detect *hisG* by RT-qPCR |
| rplO-for | ATTCGGCTTCACTTCTCGTAA | To detect *rplO* by RT-qPCR |
| rplO-rev | CTTTCAGCGTGTTCAGGTCTA | To detect *rplO* by RT-qPCR |

**SI References**

1. Liu, F.; Qimuge; Hao, J.; Yan, H.; Bach, T.; Fan, L.; et al. AspC-mediated aspartate metabolism coordinates the Escherichia coli cell cycle. *Plos One*.2014; *9* (3), e92229.

2. Casadaban, M. J. Transposition and fusion of the lac genes to selected promoters in Escherichia coli using bacteriophage lambda and Mu. *J Mol Biol*.1976; *104* (3), 541-555.

3. Ferenci, T.; Zhou, Z.; Betteridge, T.; Ren, Y.; Liu, Y.; Feng, L.; et al. Genomic sequencing reveals regulatory mutations and recombinational events in the widely used MC4100 lineage of Escherichia coli K-12. *J Bacteriol*.2009; *191* (12), 4025-4029.

4. Gon, S.; Camara, J. E.; Klungsoyr, H. K.; Crooke, E.; Skarstad, K.; Beckwith, J. A novel regulatory mechanism couples deoxyribonucleotide synthesis and DNA replication in Escherichia coli. *Embo J*.2006; *25* (5), 1137-1147.

5. Hansen, E. B.; Atlung, T.; Hansen, F. G.; Skovgaard, O.; Von, M. K. Fine structure genetic map and complementation analysis of mutations in the dnaA gene of Escherichia coli. *Mol Gen Genet*.1984; *196* (3), 387-396.

6. Datsenko, K. A.; Wanner, B. L. One-step inactivation of chromosomal genes in Escherichia coli K-12 using PCR products. *Proc Natl Acad Sci USA*.2000; *97* (12), 6640-6645.

7. Ellermeier, C. D.; Janakiraman, A.; Slauch, J. M. Construction of targeted single copy lac fusions using lambda Red and FLP-mediated site-specific recombination in bacteria. *Gene*.2002; *290* (1–2), 153-161.

8. Krause, M.; Rückert, B.; Lurz, R.; Messer, W. Complexes at the replication origin of Bacillus subtilis with homologous and heterologous DnaA protein. *J Mol Biol*.1997; *274* (3), 365.

9. Brondsted, L.; Atlung, T. Anaerobic regulation of the hydrogenase 1 (hya) operon of Escherichia coli. *J Bacteriol*.1994; *176* (17), 5423-5428.

10. Chang, A. C.; Cohen, S. N. Construction and characterization of amplifiable multicopy DNA cloning vehicles derived from the P15A cryptic miniplasmid. *J Bacteriol*.1978; *134* (3), 1141-1156.
